# Proteogenomic Features of the Highly Polymorphic Histidine-rich Glycoprotein Arose Late in Evolution

**DOI:** 10.1016/j.mcpro.2023.100585

**Published:** 2023-05-25

**Authors:** Yang Zou, Bas van Breukelen, Matti Pronker, Karli Reiding, Albert J.R. Heck

**Affiliations:** 1 Biomolecular Mass Spectrometry and Proteomics, Bijvoet Center for Biomolecular Research and Utrecht Institute for Pharmaceutical Sciences, University of Utrecht, Utrecht, the Netherlands; 2 Netherlands Proteomics Center, Utrecht, the Netherlands

**Keywords:** serum proteomics, histidine-rich glycoprotein (HRG), plasma proteomics, protein biomarker, proteogenomics, 1000-genome project

## Abstract

Histidine-rich glycoprotein (HRG) is a liver-produced protein circulating in human serum at high concentrations of around 125 μg/ml. HRG belongs to the family of type-3 cystatins and has been implicated in a plethora of biological processes, albeit that its precise function is still not well understood. Human HRG is a highly polymorphic protein, with at least five variants with minor allele frequencies of more than 10%, variable in populations from different parts of the world. Considering these five mutations we can theoretically expect 3^5^ = 243 possible genetic HRG variants in the population. Here, we purified HRG from serum of 44 individual donors and investigated by proteomics the occurrence of different allotypes, each being either homozygote or heterozygote for each of the five mutation sites. We observed that some mutational combinations in HRG were highly favored, while others were apparently missing, although they ought to be present based on the independent assembly of these five mutation sites. To further explore this behavior, we extracted data from the 1000 genome project (n ∼ 2500 genomes) and assessed the frequency of different HRG mutants in this larger dataset, observing a prevailing agreement with our proteomics data. From all the proteogenomic data we conclude that the five different mutation sites in HRG are not occurring independently, but several mutations at different sites are fully mutually exclusive, whereas others are highly intwined. Specific mutations do also affect HRG glycosylation. As the levels of HRG have been suggested as a protein biomarker in a variety of biological processes (*e.g.*, aging, COVID-19 severity, severity of bacterial infections), we here conclude that the highly polymorphic nature of the protein needs to be considered in such proteomics evaluations, as these mutations may affect HRG’s abundance, structure, posttranslational modifications, and function.

## Highlights

• The histidine-rich glycoprotein (HRG) contains five minor allele frequencies (MAFs).
• Proteogenomics probes correlations between MAFs in the human population.
• Some mutations are mutually exclusive, others preferentially co-occur.
• When studying HRG, accounting for allelic variants is crucial.
• HRG mutations appeared late in evolution and led to partial loss of *N*-glycosylation.

## Graphical Abstract

**Figure:**
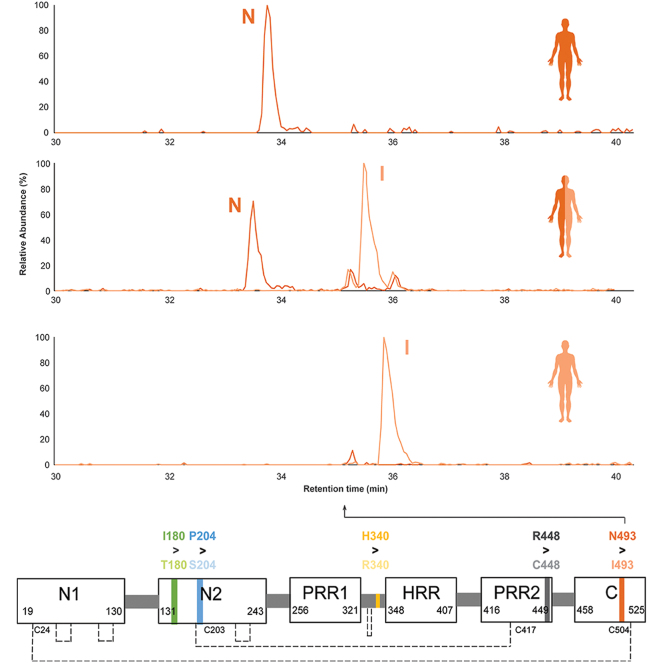

## In Brief

Histidine-rich glycoprotein (HRG) is a liver-produced protein found abundantly in human blood, exhibiting at least five minor allele frequencies (MAFs). Proteogenomics analysis on individual donors reveals that certain mutations are mutually exclusive, while others preferentially co-occur. These HRG mutations appeared late in evolution and led to partial loss of N-glycosylation. HRG mutations may affect the protein's abundance, structure, posttranslational modifications, and thus function. Therefore, when studying HRG, accounting for allelic variants is crucial.

**Fig. 1:**
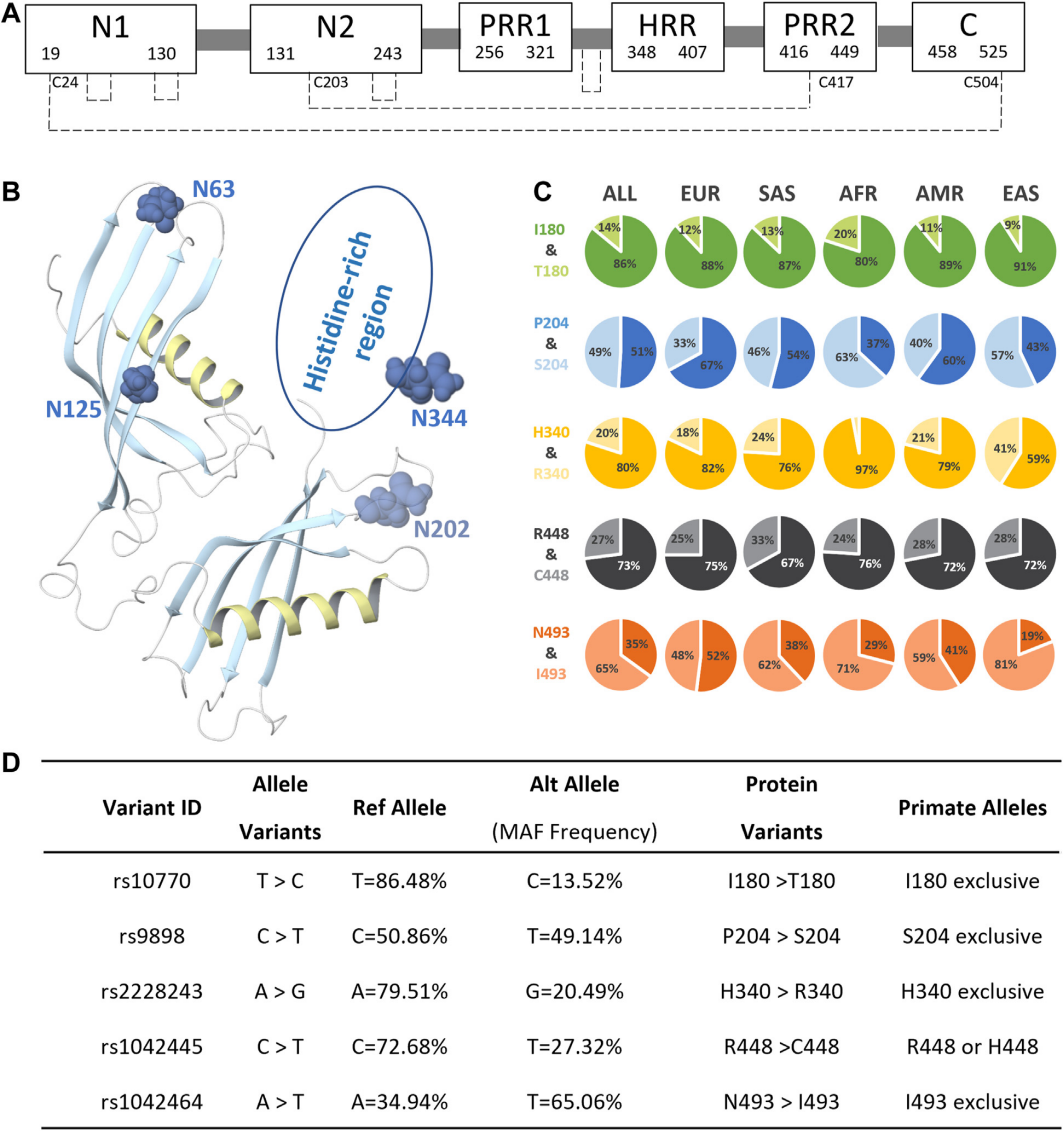
S**chematic structure of histidine-rich glycoprotein (HRG) and its gene variants with minor allele frequencies (MAFs) of more than 10%.***A*, schematic representation of protein domains of human serum HRG. HRG consists of six domains: two N-terminal domains (N1 and N2), two proline-rich regions (PRR1 and PRR2), a histidine-rich region (HRR), and a C-terminal domain (C). The disulfide bridges are depicted in dashed lines. *B*, partial structure and *N*-glycosylation sites on HRG. The structure of HRG is obtained by Swiss-model homology modeling based on Fetuin-B (6hpv.1.A) (3, 40, 41, 42, 43). The histidine-rich domain, which does not tend to crystallize, is represented with an oval. The residues of the *N*-glycosylation sites are shown as dark blue. A glycosylation site at Asn202 is induced by rs9898 (30). *C*, the frequencies of five abundant gene variants of HRG in different subpopulations. The subpopulations are named as follows: EUR, European; SAS, South Asian; AFR, African; AMR, American; EAS, East Asian. *D*, gene variants of HRG with MAF of more than 0.1 (*i.e.*, 10% of the population) and their corresponding primate alleles (38).

Next to the extremely abundant albumin and immunoglobulin proteins present in serum several other proteins are fairly abundant, among them the histidine-rich glycoprotein (HRG). HRG is a glycoprotein mostly produced in the liver that circulates in human serum at concentrations of approximately 100 to 150 μg/ml. Structurally, the HRG protein consists of 525 amino acids spanning six annotated domains: two N-terminal domains (N1 and N2 domain), two proline-rich regions (PRR1 and PRR2 domain), a histidine-rich domain (HRR domain), and a C-terminal domain (C domain) (1) (Fig. 1*A*). HRG belongs to the family of type-3 cystatins together with other fairly abundant serum proteins such as α2-Heremans Schmid-glycoprotein, fetuin-B, and kininogen (2). These four proteins are structurally and functionally related, and their genes are also located closely to each other on chromosome 3. They all share sequence and structural homology, and all contain two to three cystatin domains. Fetuin-B is even a very close paralog of HRG; alignment of these two genes reveals around 35% identity, and also structurally these two proteins are very alike (Fig. 1*B*) (2, 3). Unique for HRG are its histidine- and proline-rich regions. This unique histidine-rich domain enables efficient HRG purification from serum using Ni^2+^ or Co^2+^ metal-affinity chromatography (4, 5, 6, 7, 8, 9).

Although HRG belongs to some of the most abundant proteins in serum and is fairly well studied, the precise function of HRG in serum is still not well defined. This is most likely due to HRG’s complex multilayer molecular characteristics. To illustrate this, HRG has been reported to interact with a variety of ligands, including heparin (10), phospholipids (11), plasminogen (12), fibrinogen (13), heme (14), Zn^2+^ (15), and even more proteins and cofactors. This suggests HRG functions as an adaptor molecule that plays a role in numerous important biological processes, such as antiangiogenic activity (16, 17, 18), immune complex clearance (19), pathogen clearance (11, 20, 21, 22, 23), coagulation (24), and fibrinolysis (25).

In humans, HRG is a highly polymorphic protein. Among the human population quite a few mutations in HRG are known, of which some are highly frequent. These allele frequencies are somewhat different in the populations from different parts of the world (Fig. 1*C*). Therefore, here, unless stated otherwise, we use the reported average (26). There are five mutation sites in HRG with minor allele frequencies (MAFs) of more than 10% (Fig. 1*D*). A single mutation site can lead to three variants, with individuals being either homozygote for AA or aa, or heterozygote Aa/aA. With HRG harboring five frequently mutated sites, already 3^5^ = 243 possible genetic variants could theoretically be present in the human population. These distinct mutations may affect the structure and function of HRG. For instance, the variant occurring at residue His340Arg substitution has been associated with divergent serum HRG levels (27). The MAF occurring at residue Pro204Ser substitution is also very high but quite divergent between different populations, namely, around 33% for Ser in Europeans but 63% in African populations (Fig. 1*C*). This particular mutation has been associated with aging and also is a predictor for the risk of mortality (28). Besides, the Pro204Ser substitution has also been associated with activated partial thromboplastin time, which is associated with risk of thrombosis and coagulation disorders (29). Moreover, this Pro204Ser substitution is in proximity of Asn202, a site that may become *N*-glycosylated, but only when a Ser204 is present (30).

Here, we set out to purify HRG from about 50 μl of serum originating from 44 individual donors. Using shotgun proteomics, we were able to achieve good sequence coverage, including peptides that covered all the five distinct mutation sites. Through these measurements we were able to define the HRG mutations present at each of the five mutation sites for each donor and whether they were either homozygote or heterozygote for that site. We used these data to investigate a potential relationship between all these five mutations. Investigating first a possible pairwise interplay between two mutation sites, we observed that the occurrence of some mutations at certain sites were mutually exclusive, whereas others apparently strengthened each other. To validate these findings, we next compared our proteomics data with data from the 1000 genome project and monitored the co-occurrence of these five mutations in genomes of n ∼2500 humans. The proteomics and genomics data were found to be in good agreement, therefore supporting the notion that the five mutation sites cannot be considered as independent events. We were also able to confirm that the Pro204Ser substitution leads, when Ser is present, to an additional *N*-glycosylation site in HRG at Asn202. All these observations display the wealth of genotypes and glycoproteoforms HRG may possess in human serum. Therefore, we argue, when considering HRG as a putative serum protein biomarker (31, 32, 33), this fine-tuning in its natural proteogenomics occurrence needs to be accounted for.

## Experimental Procedures

### Study Cohort

The 44 serum samples were obtained from two prior-gathered cohorts from Amsterdam (NL) and Bologna (Italy). These serum samples were obtained in accordance with the ethics of the board of Sanquin (Amsterdam, the Netherlands) and Comitato Etico di Area Vasta Emilia Centro (Bologna, Italy) and following the Declaration of Helsinki principles. All donors had given their written informed consent. The serum samples from Italy were from a cohort that is registered at www.clinicaltrials.gov with the identifier NCT04343053. Whole blood from each individual donor was collected in a vacuette tube (Greiner Bio-One) containing Z serum clot activator. The whole blood was left undisturbed at room temperature for 30 to 60 min before removal of the clotted material by centrifugation at 1800*g* for 20 min. Individual serum samples were transferred to 1.5-ml Eppendorf tubes as 1-ml aliquots, snap frozen in liquid nitrogen, and stored at −80 °C.

### HRG Protein Purification

HRG was isolated from human serum using immobilized metal affinity chromatography (IMAC) using a cobalt-loaded resin (Thermo Scientific). Briefly, cobalt slurries were washed with 3 × binding buffer (supplemental Table S1). A volume of 50 μl of beads was incubated on a tube revolver with 100 μl serum diluted with 1000 μl binding buffer for 3 h at 4 °C. Then the beads were subsequently washed with washing buffer 1 to 7 (supplemental Table S1). The purified HRG protein was eluted with eluting buffer twice (supplemental Table S1). The supernatant was combined and vacuum dried. Imidazole, which was used for the buffer, was purchased from Sigma-Aldrich.

### In-solution Proteolysis

Purified HRG was resuspended in 100 mM Tris-HCl and then mixed with digestion buffer containing 200 mM Tris-HCl (pH 8.5), 2% *w/v* sodium deoxycholate (SDC), 10 mM tris(2-carboxyethyl)phosphine, and 60 mM chloroacetamide. The sample was denatured at 95 °C for 10 min and then incubated in the dark for 45 min. The resulting peptide mixtures were further digested overnight at 37  °C by trypsin (1:30; *w/w*). On the next day, SDC was removed by acid precipitation using 0.5% trifluoroacetic acid. The peptides were desalted by using an Oasis PRiME HLB plate (Waters), then dried and stored at −80  °C. Tris-HCl, tris(2-carboxyethyl)phosphine, chloroacetamide, SDC, trifluoroacetic acid, and trypsin were purchased from Sigma-Aldrich.

### Bottom-Up Proteomics

Shotgun liquid chromatography (LC)–tandem mass spectrometry (MS/MS) was performed by using an UltiMate 3000 HPLC system (Thermo Fisher Scientific) coupled to a Orbitrap Exploris Mass Spectrometer (Thermo Fisher Scientific). About 250 ng of the digested peptides were loaded onto a 2-cm trap column (in-house packed with ReproSil-Pur C18-AQ, 3 μm) (Dr Maisch GmbH) coupled to a 50-μm-inner-diameter 50-cm analytical column (in-house packed with Poroshell 120 EC-C18, 2.7 μm) (Agilent Technologies). As for gradient separation, 0.1% formic acid (*v/v*) was used as the mobile phase A, while 0.1% formic acid in acetonitrile (*v/v*) was mobile phase B. At the first stage, the mobile phase increased from 9% B to 13% for 1 min, from 13% to 44% in the next 40 min, from 44% to 99% for 3 min, after which it was maintained at 99% for 4 min. Afterward, B decreased to 9% in 1 min and was maintained at 9% for 10 min. The flow rate was set as 300 nl/min. Peptides were ionized using a spray voltage of 2 kV in combination with an ion transfer capillary that was heated to 275 °C. The mass spectrometer was set to acquire full-scan mass spectra (*m/z* 350–2000) for a maximum injection time of 50 ms at a mass resolution of 120,000. Up to 15 of the most intense precursor ions were selected for MS/MS. Higher-energy collisional dissociation (HCD) MS/MS (*m/z* 120–4000) acquisition was performed in the HCD cell, with the readout in the Orbitrap mass analyzer at a resolution of 60,000 and a maximum injection time of 50 ms with a normalized collision energy of 29%. For the product-dependent-stepping-HCD fragmentation, the collision energy was 10%, 25%, and 40%.

### Proteomics Database Search and Data Analysis

As we expected both nonmodified and glycopeptides, we analyzed the raw files using Byonic v4.3.4 (PMi). MS/MS spectra were searched against the full annotated human proteome (Swiss-Prot, release date July 2021, 20,398 entries), complemented with all five dominant mutations of HRG. The search parameters were fixed modification of cysteine residues (+57 Da), variable modification of methionine oxidation (+16 Da), and full trypsin cleavage, with at most six missed tryptic cleavages, 10 ppm error tolerance in MS, and 20 ppm error tolerance in MS/MS. False discovery rates were <1%. When tryptic peptides were detected covering mutation sites, Skyline (v3.7.0.11317) was used to perform a relative quantification. Doing this, we integrated each variant of the peptides that was reported by Byonic, which included all major miscleavages and oxidation variants. Integrations obtained as such were subsequently curated to adhere to the following criteria: (1) ≤ 5 ppm error to the theoretical mass, (2) having an idotp of ≥80% with the theoretical isotopic pattern, (3) eluting within ± 3.5 min of the mean retention time for that peptide, and (4) having no apparent overlapping isotopic patterns.

### Thousand Genome Analysis

To compare our observed proteomics data on the different alleles and combinations of alleles we obtained the 1000 genome data (1000 genome database, release date October 2015) (34), including sample ID, genotype, genotype frequency, population(s), and allele counts, from ENSEMBL database (35) for each variant. All variants were combined per sample ID to obtain a comprehensive overview of all allele combinations per individual. Subsequently, we counted the observed allele combinations and from this calculated their combined frequencies.

### Phylogenetic Context

For each allele we looked at the phylogenetic context, using the 90 eutherian mammals EPO-Extended dataset from ENSEMBL. From these alignments we determined which allele is observed for which species (*e.g.*, primates or nonprimates) and where it starts to differ, if at all, from the human reference allele. This information was subsequently used to obtain an indication on how well this allele is conserved and if it was mutated in the relative early or far past (35).

### Polyacrylamide Gel Electrophoresis

A total of 15 μl sample was mixed with loading buffer for protein analysis. The sample was then heated to 95 °C for 5 min. The samples and SDS-PAGE standards (Bio-Rad) were loaded into SDS-PAGE gel wells. Electrophoresis was carried out at 160 mA for 1 to 2 h until the standards properly separated. The gel was then removed and put in the appropriate volume Coomassie G-250 stain solution (Bio-Rad), ensuring that the gel was fully covered by staining solution and placed on a horizontal shaker. The staining solution was poured out, and the gel was washed with water for 4 to 24 h. Water was replaced 3 to 5 times during the period until the blue background was almost removed, enhancing the dyeing effect on the protein bands.

## Results and Discussion

### Characterization of Allelic Frequencies in Serum Proteomics Data

For our proteomics analyses HRG was purified from 44 individual serum samples, obtained from donors, with consent, originating from different earlier gathered cohorts, with all donors being from European origin (*i.e.*, Netherlands and Italy, see Experimental Procedures for more details). Making use of its unique histidine-rich domain, HRG could be isolated from serum by IMAC using a cobalt-loaded resin (see Experimental Procedures). After purification of HRG from individual serum samples, we digested HRG with trypsin. In all the 44 serum samples, we carefully inspected the shotgun proteomics data for the appearance of tryptic peptides covering each of the individual five mutations. Both Byonic and Skyline were used to analyze the proteomics data, to provide qualitative and quantitative insight into the presence and relative abundance of the five distinct allele variants in HRG purified from each of the donors. We obtained a typical sequence coverage of around ∼75%, and most of the time we detected tryptic peptides covering each of the five mutation sites.

**Fig. 2:**
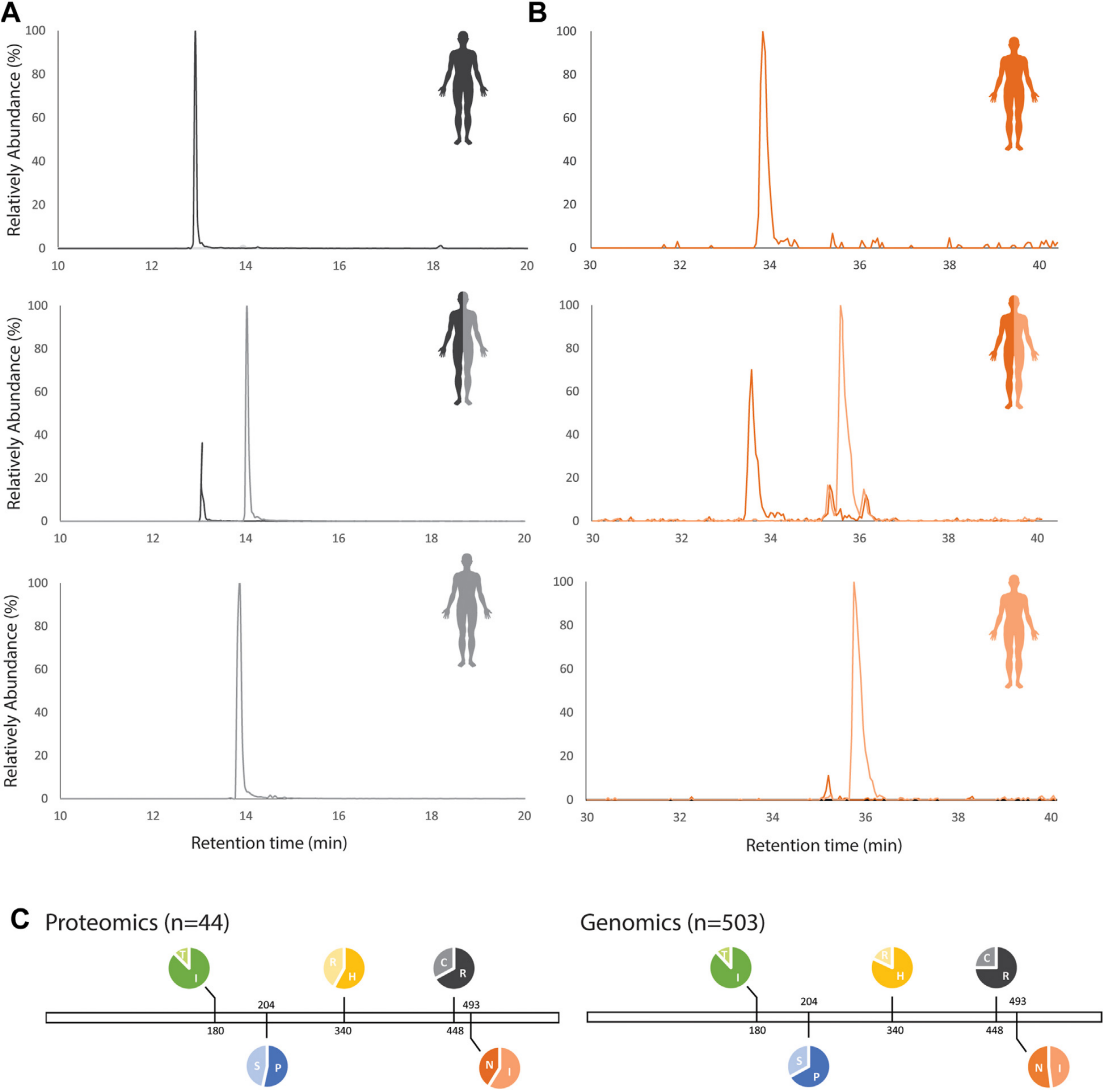
**Gene variants of histidine-rich glycoprotein (HRG) measured by proteomics.** Prototypical LC-MS traces of unique allele-specific peptides detected in serum HRG purified from different donors (Skyline was used for allele classification and quantification) of (*A*) the Arg448Cys substitution and (*B*) and the Asn493Ile substitution. The spectra from top to bottom are the LC-MS traces for donors being homozygote AA, heterozygote Aa/aA, or homozygote aa. *Dark gray*, Arg448; *light gray*, Cys448; *dark orange*, Ile493; *light orange*, Asn493. *C*, the frequencies of gene variants of HRG with minor allele frequencies of more than 10% as observed in the proteomics data from the 44 donors (*left*) and (for comparison) as extracted from the European genomics data from the 1000 genomes project (*right*).

Since humans are diploid organisms, individuals can be homozygous or heterozygous for genetic variations. Therefore, in heterozygote donors we would expect distinct peptides harboring either the dominant or alternative mutant. In Figure 2, illustrative prototypical LC-MS traces are depicted of allele-specific peptides of HRG, obtained from homozygote or heterozygote donors carrying either the Arg448Cys substitution (Fig. 2*A*) or the Asn493Ile substitution (Fig. 2*B*). The whole dataset of alike LC-MS traces of peptides, covering all mutations for all donors, are provided in supplemental Figs. S1–S10. In addition, MS/MS fragmentation spectra, explicitly assigning the different mutations, are provided in supplemental Fig. S11. Nearly for each mutation our cohort represented donors being either AA or aa homozygote or Aa/aA heterozygote. In case of homozygotes, we counted the allele occurrence twice (for each of the two genes), which allowed us to calculate the frequency of occurrence of each mutation in our cohort of 44 donors. A summary of all data for each mutation in HRG in our cohort of 44 people is provided in Supplementary Data Excel file 1.

In more detail, in our n = 44 cohort proteomics dataset we observed a ratio of 88%:12% for the Ile180Thr substitution rs10770 (T > C), a ratio of 53%:47% for the Pro204Ser substitution rs9898 (C > T), a ratio of 60%:40% for the His340Arg substitution rs2228243 (A > G), a ratio of 72%:28% for the Arg448Cys substitution rs1042445 (C > T) with Arg>Cys, and for Asn493Ile substitution rs1042464 (A > T) a ratio of 41%:59%, with Ile dominant (Fig. 2*C*). Although our (fully European) cohort of 44 is relatively small, these ratios are relatively close to the expected MAFs within the European cohort based on data from the 1000 genome project (Fig. 2*C*). Based on this agreement we argue that the proteomics-based measurement of allelic frequencies occurring in HRG purified from serum of individual donors by IMAC-based chromatography is feasible.

### Pairwise Co-occurrences of Allele-specific Mutations in the Proteomics Data

**Fig. 3:**
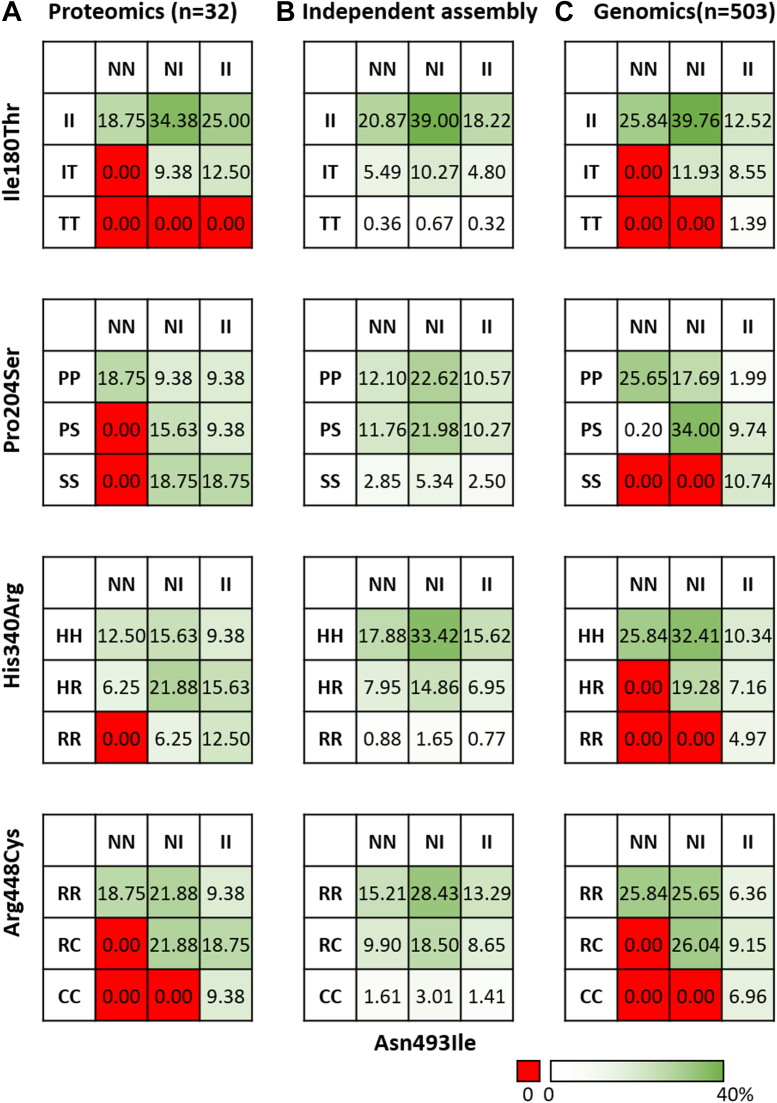
**Pairwise co-occurrences of allele-specific mutations in histidine-rich glycoprotein between position 493 and other mutation sites.***A*, frequencies observed in the proteomics data (n = 32). *B*, theoretical frequencies of co-occurrences based on independent assembly. *C*, frequencies observed in the European subset from the 1000 genomes project.

Although our cohort is rather small (n = 44), several striking features could be observed in the proteomics data, especially in the co-occurrence of specific mutations. We first focused on pairwise co-occurrences. For instance, donors found at the peptide level to be homozygote in Pro204 (*i.e.*, no peptides were detected with a Ser204) also never presented an HRG peptide harboring an Ile on position 493, but exclusively always an Asn493. From this, one may infer that all the donors being homozygote at Asn493 should also be homozygote at Pro204. Similarly, in HRG of donors wherein we exclusively detected peptides with an Asn493 (and not Ile493), we also only detected allele-specific peptides carrying an Arg448 (and not Cys448). From this, one may argue that all the homozygote Asn493donors should also be homozygote in Arg448. To understand these pairwise correlations, we next accumulated from the proteomics data all pairwise co-occurrences of mutations between position 493 and other positions (Fig. 3*A*). We also calculated the expected pairwise co-occurrences when all mutations would be considered as independent events (based on the European single MAFs) (Fig. 3*B*). From the data depicted in Figure 3, *A* and *B* it becomes apparent that most pairwise combinations do not fulfill the expectancy based on independent assembly, with some pairwise combinations being highly favored and others being absent, even when these should be present based on the independent assembly model. Of note, not in all 44 HRG samples we were able to obtain data on each mutation site with sufficient evidence to extract information on the allotype, either due to low intensities or mismatches with expected isotope ratios (supplemental Figs. S2, S5, S6, S9 and S10). Therefore, just 32 samples met our stringent filtering conditions and were used for the data analysis. Table 1 illustrates the pairwise combinations that should be observed, considering both homo- and heterozygote occurrences. If both mutations would be independent, the frequencies of the pairwise combinations can be calculated by multiplying the MAFs of the single mutants.

**Table 1:** Co-occurrence analysis of mutations

	BB	Bb	bb
AA	AA × BB	AA × Bb	AA × bb
Aa	Aa × BB	Aa × Bb	Aa × bb
aa	aa × BB	aa × Bb	aa × bb

When considering two mutations, there can be nine pairwise combinations, where A represents the first allele and B represents the second allele.

Clearly, the proteomics data reveal that, of the five mutation sites in HRG, some do show a strong correlation in co-occurrence. However, this finding is based on a relatively small number of donors. Moreover, shotgun proteomics analysis remains a stochastic process, and thus certain peptides may not be consistently detected. On the other hand, since we do observe unique highly abundant peptides of each single mutation occurring in the five mutation sites in multiple samples, we believe that, when these peptides are not detected in samples from other donors, they are either very low abundant or not present.

To further examine the findings that pairwise mutations do not assemble independently, we charted the data encompassed in the 1000-genomes project, which is actually an accumulation of 2504 fully sequenced human genomes, and compared in a pairwise fashion how often mutations in HRG co-occurred (34, 36). We used the reported frequencies of the European cohort within the 1000 genome project (n= 503, phase 3), whereby these frequencies were obtained from the ENSEMBL database (36). These extracted data are provided in Supplementary Data Excel file 2. The result of this pairwise analysis is displayed in Figure 3*C*. At first glance the agreement between the proteomics data on the small cohort (n = 32) and the 1000 genome data, from the 503 (European) full genomes, is very good, especially when compared with the data produced from the independent assembly model. Several pairwise mutations predicted to occur rather frequently based on the independent assembly model, for instance, homozygote Ser204 with homozygote at Asn493, are completely missing in both the proteomics and genomics data. Thus, from the experimental proteomics and genomics data presented in Figure 3, it becomes clear that in HRG pairwise combinations of the five mutations do not occur by independent assembly of single site mutations, with some pairwise combinations highly favored while others seem not to be present.

Although the proteomics data and genomics data show overall great similarity, we also observed evidence for a few seemingly co-occurring mutations in the proteomics data that were absent in the genomics data. For instance, we do find two donors who are heterozygote in His340Arg and homozygote in Asn493Asn, as well as two donors who are homozygote in Arg340Arg and heterozygote in Asn493Ile combinations in our proteomics data (Fig. 3*A*), which are not observed in the 1000 genomes dataset (Fig. 3*C*). Presently, we cannot explain this apparent discrepancy but do provide the apparently robust evidence of these co-occurring mutations in supplemental Figs. S12–S14.

### Total Number of HRG Genetic Variants Present in the Human Population

**Fig. 4:**
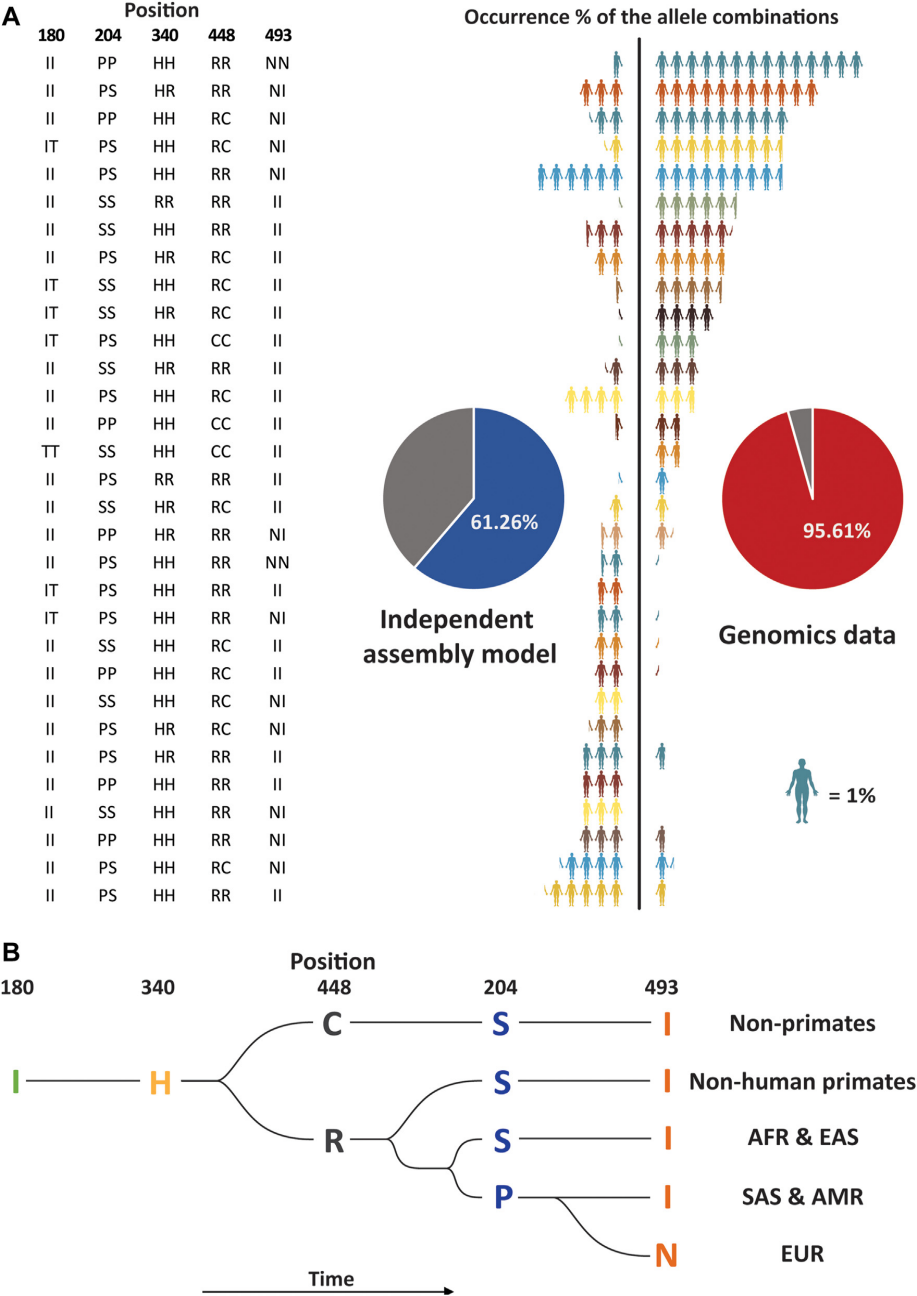
**Theoretical (independent assembly) and experimental (1000 genome data) co-occurrences of all five allele frequencies in histidine-rich glycoprotein (HRG).***A*, occurrence percentage of the top 20 abundant combinations based on an independent assembly model (*left*) and the experimental 1000 genome data (*right*). The *y*-axis depicts the combinations of most frequent gene variants within HRG, with pairwise amino acids at position 180, 204, 340, 448, and 493. Cartoon figures with different colors represent these different allele combinations. Each figure represents 1% of the population. The pie chart insets depict the summed relative contribution of the 20 most frequently calculated/observed combinations. *B*, proposed evolutionary tree of the five mutations in HRG, enlightening why certain combinations of co-occurrences are unlikely to be observed. Each branch represents the most abundant allele combination of HRG s in different regions.

Next, we extended our analysis beyond these pairwise combinations of mutations, calculating the frequency of occurrence of combinations of all five mutations in the human population. To reiterate, a single mutation site can lead to three variants, with donors being either homozygote for AA or aa, or heterozygote Aa/aA. With HRG harboring five mutation sites, 3^5^ = 243 genetic variants of HRG could theoretically be present in the human population. We calculated, similar as we did in the pairwise analyses, the expected frequency of occurrence of all these 243 possible combinations when these five mutations would assemble independently from each other. In addition, from the full 1000 genome dataset (n = 2504), we counted the frequency of occurrences of each of these 243 combinations. The outcome of this analyses is depicted in Figure 4*A*. Interestingly, only about 50 combinations (of 243) dominate the frequency-of-occurrence spectrum in the genomics data, several of which seem to be extremely enriched when compared with what would be expected based on the independent assembly model. Therefore, also from this analysis it becomes clear that the independent assembly model delivers substantial discrepancies when compared with the experimental genomics and proteomics data.

To further highlight these differences, we also depict the frequency of occurrence of the top 20 most abundant allelic combinations of the five mutation sites considering both the independent assembly model (Fig. 4*A*, left pie chart) and the experimental human genome data (Fig. 4*A*, right pie chart). They only share nine of these two top-20 combinations of the five mutation sites. In frequency the top 20 makes up ∼61% of all occurrences according to the independent assembly model, while the top 20 in the 1000 genome data makes up ∼96% of all occurrences. Thus, of 3^5^ = 243 combinations only a few dozen dominate the palette of allelic occurrences.

The allelic occurrence of the fully homozygote II_PP_HH_RR_NN combination is the most abundant in the 1000 genome data, present in ∼14% of the tested human population. This is a very strong enrichment when compared with the independent assembly model, which predicts that less than 1% of the population should carry this combination. In addition, many combinations predicted to be frequent based on the independent assembly model do not occur in the 1000 genome data set. For instance, and as already described in the pairwise comparison section, all allelic combinations that comprise a homozygote Ser204 with a homozygote Asn493 are completely missing in the 1000 genome data.

To investigate whether this is a unique phenomenon for HRG, we performed a similar analysis on the human serum protein alpha-1-antitrypsin (A1AT) that also harbors several well-known mutations (37). However, for A1AT we found that the distribution of allelic variants were much more similar between the prediction based on the independent assembly model and the experimental 1000 genome data (n = 2504) (37). All data related to the analyses on the allelic frequencies of HRG and A1AT are provided in Supplementary Data Excel files 3 and 4, respectively, and supplemental Fig. S15. In addition, we also compared the European allelic variant distribution from HRG and A1AT between the independent assembly model and the (smaller) European subcohort from the 1000 genome project (n = 503), provided in Supplementary Data Excel files 5 and 6 and supplemental Figs. S16 and S17, respectively. Also, by using this smaller subcohort, we did observe similar discrepancies between the independent assembly model and the genome data, again much more so for HRG than for A1AT. Currently, we lack a good explanation for these distinct proteogenomic features for human HRG and A1AT.

### Enriched Observed HRG Genotypes Are Consequences of Evolutionary Divergence

We attempted to address the question why some of the 243 possible HRG genotypes are enriched, diminished, or not present at all. We first queried whether some of these five mutations might have originated late in evolution and in distinct parts of the world. Such a hypothesis may be tested from the data summarized in Figure 1*C*. For instance, compared with the global average, Ser204 is much more prevalent in the African and East Asian population, whereas Pro204 is most dominant in the European and American populations. Also, nearly all Africans carry exclusively His340 (∼97%), whereas especially in the East Asian population this site has a high frequency of occurrence (41%) of Arg340. Third, Europeans have relatively high frequencies of Asn493 (52%) compared with some other populations (*e.g.*, 19% in East Asians). This analysis shows that the distributions of variants of the HRG is highly affected by the origin of the cohort, which also implies that, when suggesting/testing HRG as a protein biomarker in serum proteomics studies, the origin of the cohort needs to be accounted for.

Next, we aimed to define the “ancestral” variants of HRG defined by genomics data on HRG of nonhuman primates as well as nonprimates (38). To start with the latter, when performing a BLAST search of human HRG against nonhuman primates, we found that in all these sequences no variants were observed for four of the five mutations we discussed here. In more detail, nonhuman primates have all highly conserved Ile180, Ser204, His340, and Ile493 (Fig. 1*D*). Only for the Arg448His substitution in HRG, several nonhuman primates carried either Arg or His. It seems therefore that most of the mutations in human HRG discussed here have occurred very late in evolution. Ile180 as well as His340 are conserved in mammals (Fig. 4*B*). After the beginning of the mutation on position 448, Arg448 became dominant in primates, while Cys448 is dominant in nonprimates. This particular mutation is interesting as Cys448 could potentially be involved in a disulfide bridge or provide HRG with a free Cys. Our shotgun proteomics data unfortunately do not enable us to provide evidence for either case.

The HRG variant Pro204 mainly occurred in the European, American, and South Asian populations, while Ser204 is largely conserved in the African and East Asian populations. Of the five mutations considered here, the latest mutation occurred at position 493. Ile493 has become the major allele in all populations except for the Europeans (Fig. 4*B*). This may also hint at a wider diversity of HRG genes and proteins in humans than in nonhuman primates. From this analysis it appears that most of the mutations in human HRG discussed here originated only recently and only in the human population.

Initially, when we had only considered the proteomics data, we also explored an additional rationale addressing why some of the allelic variants are potentially never observed as HRG proteins in blood, namely, that it could be the case that a certain allelic variation at the gene level would not lead to a viable expression of the protein. To test this hypothesis, we recombinantly expressed in human HEK293 cells four variants of HRG testing all possible pairwise mutations for position 204 and 493. We accordingly expressed HRG with PP204_II493, PP204_NN493, SS204_II493, and SS204_NN493. According to the proteomics and 1000 genome data SS204_NN493 HRG does not occur in nature. The expression of all these variants in the HEK293 cells led in all cases to high and, more importantly, equal levels of HRG demonstrating that the nonnatural variants are equally viable (supplemental Fig. S18).

### Functional Consequences of Specific HRG Genotypes

Our proteogenomics analysis demonstrates that, when analyzing HRG in serum proteomics studies, there is more to this than meets the eye when just considering protein levels without taking the highly polymorphic nature of HRG into account. Evidently several genome-wide association studies have taken these HRG mutations into account, and from such studies the Pro204Ser substitution, for instance, has been proposed to be associated with aging and also as a predictor for the risk of mortality following bacterial infections (28) and increased risk of thrombosis and coagulation disorders (29). This Pro204Ser substitution is of particular interest, also at the protein level. Ser204 is downstream of Asn202, and it has been reported that this Asn202 site may become *N*-glycosylated, but only when a Ser204 is present (30). In our analysis we could confirm this additional *N*-glycosylation in two separate experiments. First of all, following the recombinant expression of the four HRG variants we observed strikingly that the variants that contained the Ser204 eluted on the gel at higher apparent molecular weight than the two variants containing a Pro204 (supplemental Fig. S18). The difference in elution is likely induced by the additional *N*-glycosylation on Asn202 when Ser204 is present. Second, in our proteomics data we did observe experimental evidence for abundant and extensive glycosylation on Asn202, but only in the two recombinant samples that contained the Ser204 site. Considering that Ser204 is likely the ancestral mutation, it seems that the Pro204 mutation, likely first seen in the European population, led to a partial loss of *N*-glycosylation in humans when compared with nonhuman primates. It may therefore be of interest to compare, at the protein and functional levels, HRG from humans with its variant obtained from nonhuman primates. Thus, the Pro204Ser substitution in HRG is genetically of interest as it shows a great discordance in frequency of occurrence not only among different populations but also from a glycoproteomics point of view as the mutation leads to a loss of a *N*-glycosylation site that seems at least partially occupied when there is a Ser at position 204.

## Concluding Remarks

At the proteogenomics level, quite some intriguing variation in HRG exists across the human population. Growing evidence suggests that such mutations may affect not only the function of HRG but possibly also its expression level as well as its serum abundance and half-life (28). In addition, the highly polymorphic nature of HRG makes it more difficult to study it *in vitro* and *in vivo*, as, to extract the right conclusions about the protein’s role and function, it needs to be stated which variant was used. Especially in serum proteomics studies of large cohorts the high variability in human HRG needs to be accounted for: only 14% of individuals are homozygote for the protein sequence that can be considered as “wild type,” a number that will furthermore differ between study populations. Therefore, we conclude that abundance data should not be averaged out in large cohort studies, instead the nature of the allelic and proteoform variants should be accounted for.

## Data Availability

The mass spectrometry proteomics data have been deposited to the ProteomeXchange Consortium *via* the PRIDE (39) partner repository with the dataset identifier PXD040914. This article contains supplemental data: supplemental Figs. S1–S18, supplemental Table S1, and Supplementary Data Excel files 1–6.

## Supplemental data

This article contains supplemental data.

## Conflict of interest

The authors declare no competing interests.

## Supplementary Data

Supplemental Data

Supplementary Data Excel file 1

Supplementary Data Excel file 2

Supplementary Data Excel file 3

Supplementary Data Excel file 4

Supplementary Data Excel file 5

Supplementary Data Excel file 6
