# Supplementary material for: Proteogenomic Features of the Highly Polymorphic Histidine-rich Glycoprotein Arose Late in Evolution
Source: Mol Cell Proteomics. 2023 May 25;22(7):100585. doi: 10.1016/j.mcpro.2023.100585 (PMC10388577; doi:10.1016/j.mcpro.2023.100585)
Supplement: Supplemental Data [file mmc1.pdf]

Supplementary Material to

Proteogenomic Features of the Highly Polymorphic Histidine-rich Glycoprotein (HRG) Arose Late in Evolution

Yang Zou[1,2], Bas van Breukelen[1,2], Matti Pronker[1,2], Karli Reiding[1,2] and Albert J.R. Heck[1,2*]

[1] Biomolecular Mass Spectrometry and Proteomics, Bijvoet Center for Biomolecular Research and Utrecht Institute for Pharmaceutical Sciences, University of Utrecht, Padualaan 8, 3584 CH Utrecht, the Netherlands

[2] Netherlands Proteomics Center, Padualaan 8, 3584 CH Utrecht, the Netherlands

Supplementary Data Excel files

Supplementary Data Excel file 1. Overview of the by proteomics-determined occurrences of amino acids corresponding to the 5 HRG mutations in the proteomics cohort (n=44). - = when insufficient or ambiguous evidence was observed.

Supplementary Data Excel file 2. Overview of the nucleic acids corresponding to the 5 mutation sites, as determined by genome sequencing within the European cohort of the 1000 genome project (n= 503, phase 3).

Supplementary Data Excel file 3. Overview of the mutation combination of HRG in the independent assembly model and the 1000 genomics data (n=2504).

Supplementary Data Excel file 4. Overview of the mutation combination of A1AT in the independent assembly model and the 1000 genomics data (n=2504).

Supplementary Data Excel file 5. Overview of the mutation combination of HRG of European in the independent assembly model and the 1000 genomics data (n=503).

Supplementary Data Excel file 6. Overview of the mutation combination of A1AT of European in the independent assembly model and the 1000 genomics data (n=503).

Supplementary Table

Supplementary Table 1. Overview of buffers used in the purification of human HRG from serum.

Solution	Solution constituents/pH	Volume (μL)
Binding buffer	25 mM HEPES, 500 mM NaCl, pH 7.8	1000
Washing buffer 1	15 mM imidazole, 25 mM HEPES, 500 mM NaCl, pH 7.8	1000
Washing buffer 2	15 mM imidazole, 25 mM HEPES, 500 mM NaCl, pH 7.8	1000
Washing buffer 3	20 mM imidazole, 25 mM HEPES, 500 mM NaCl, pH 7.8	1000
Washing buffer 4	35 mM imidazole, 25 mM HEPES, 500 mM NaCl, pH 7.8	1000
Washing buffer 5	35 mM imidazole, 25 mM HEPES, 500 mM NaCl, pH 7.8	1000
Washing buffer 6	40 mM imidazole, 25 mM HEPES, 500 mM NaCl, pH 7.8	1000
Washing buffer 7	40 mM imidazole, 25 mM HEPES, 500 mM NaCl, pH 7.8	1000
Eluting buffer	500 mM imidazole, 25 mM HEPES, 500 mM NaCl, pH 7.8	100

Supplementary Figures

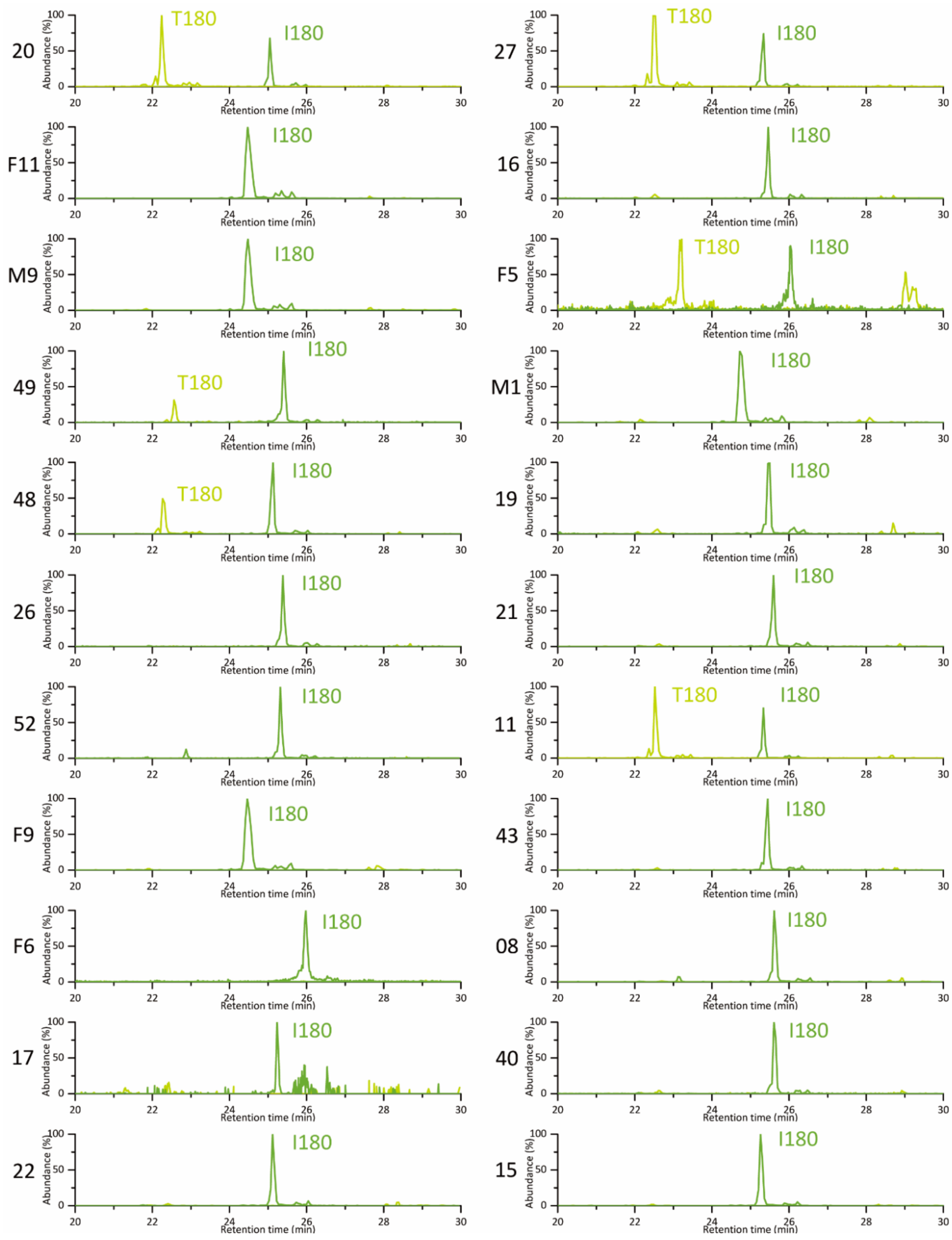

Supplementary Figure 1. LC-MS traces of unique allele specific peptides detected in HRG from the first 22 donors in order of measurement. Skyline was used for allele classification and quantification of the Ile180Thr substitution. Dark green: Ile180, light green: Thr180. The number on the left of y-axis provides the donor code.

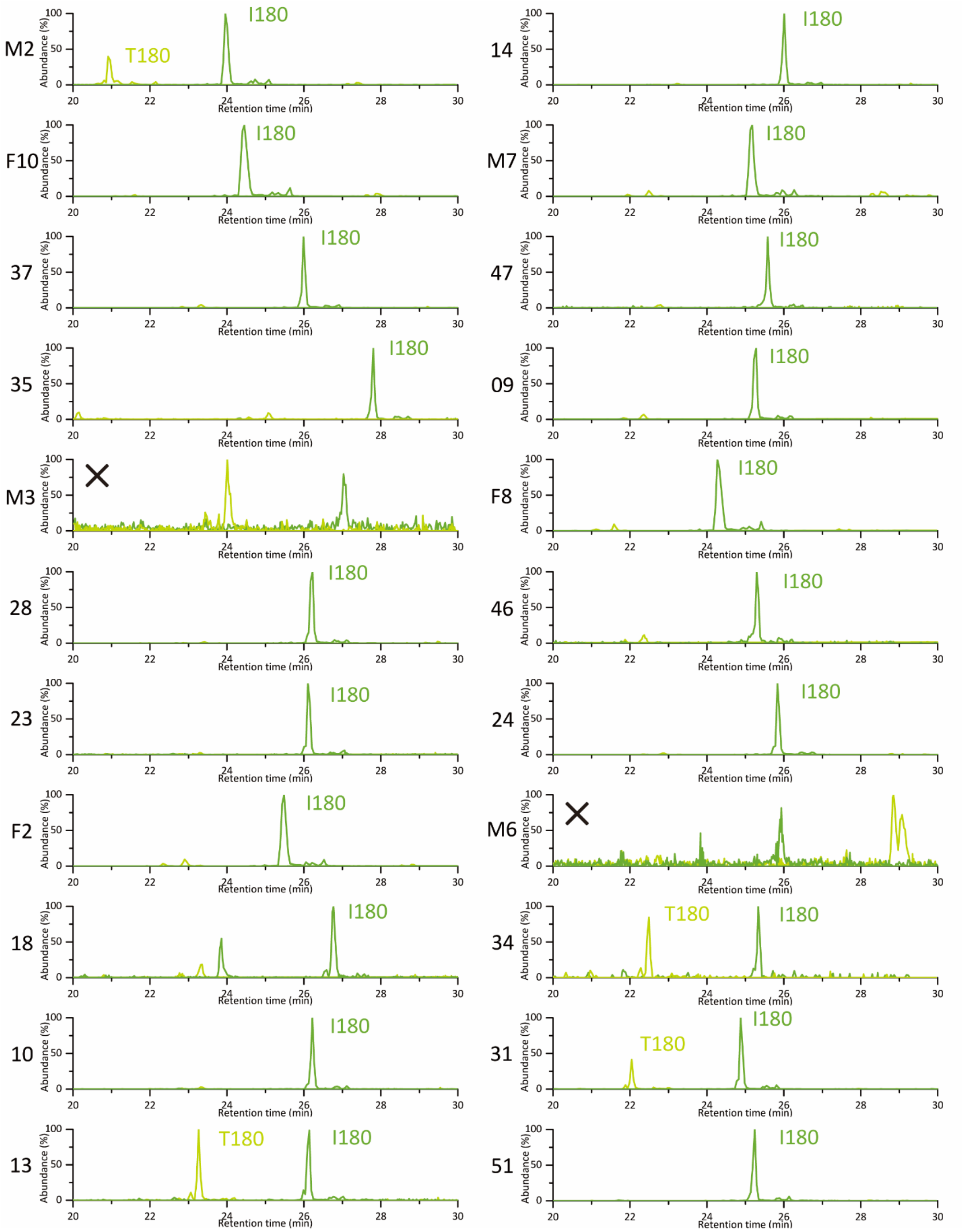

Supplementary Figure 2. LC-MS traces of unique allele specific peptides detected in HRG from the last 22 donors in order of measurement. Skyline was used for allele classification and quantification of the Ile180Thr substitution. Dark green: Ile180, light green: Thr180. The number on the left of y-axis provides the donor code. The samples with black crosses are not assigned as alleles, either due to low intensities or mismatches with expected isotope ratios.

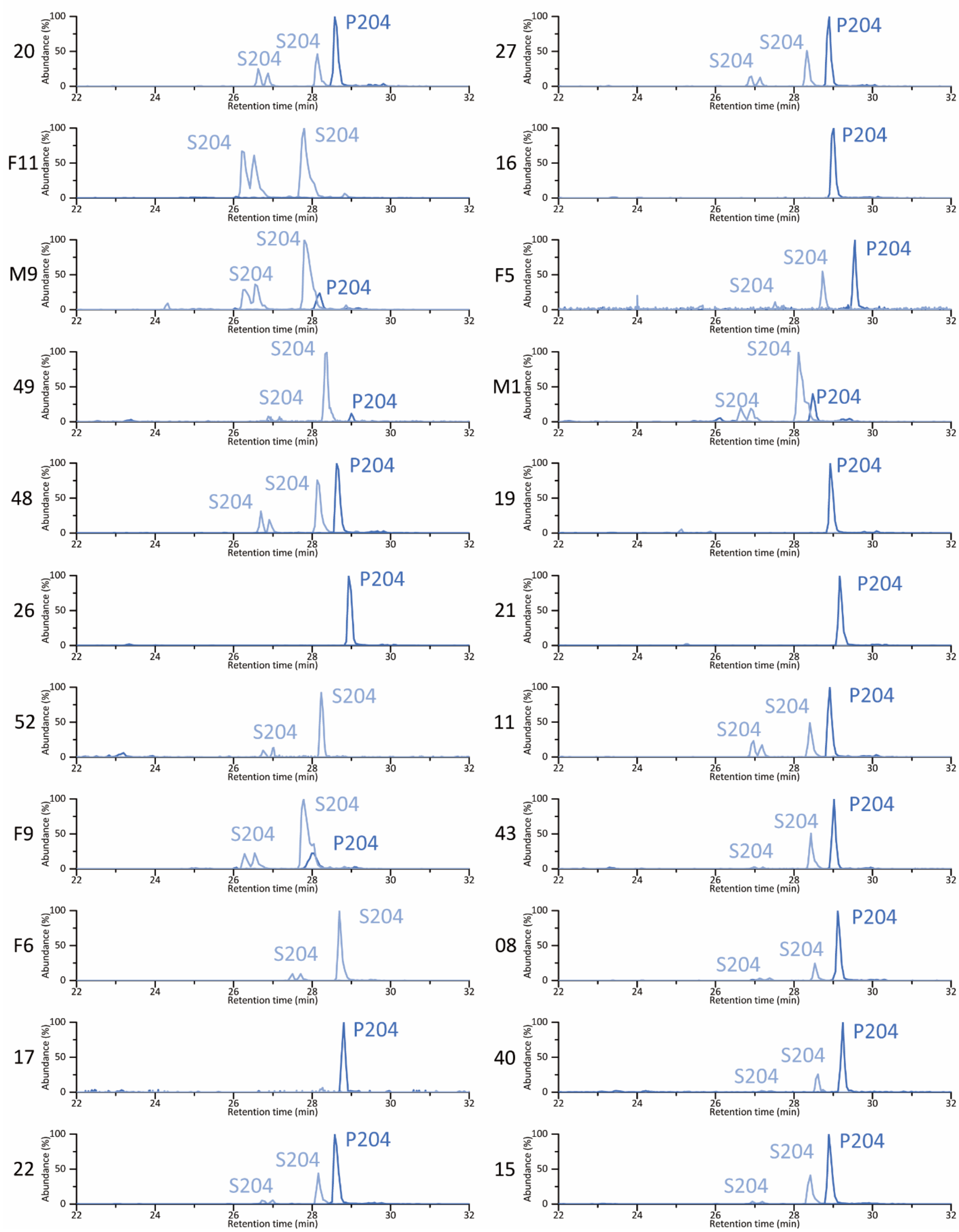

Supplementary Figure 3. LC-MS traces of unique allele specific peptides detected in HRG from the first 22 donors in order of measurement. Skyline was used for allele classification and quantification of the Pro204Ser substitution. Dark blue: Pro204, light blue: Ser204. The multiple peaks seen at Ser204 are glycoforms of the same peptide. The number on the left of y-axis provides the donor code.

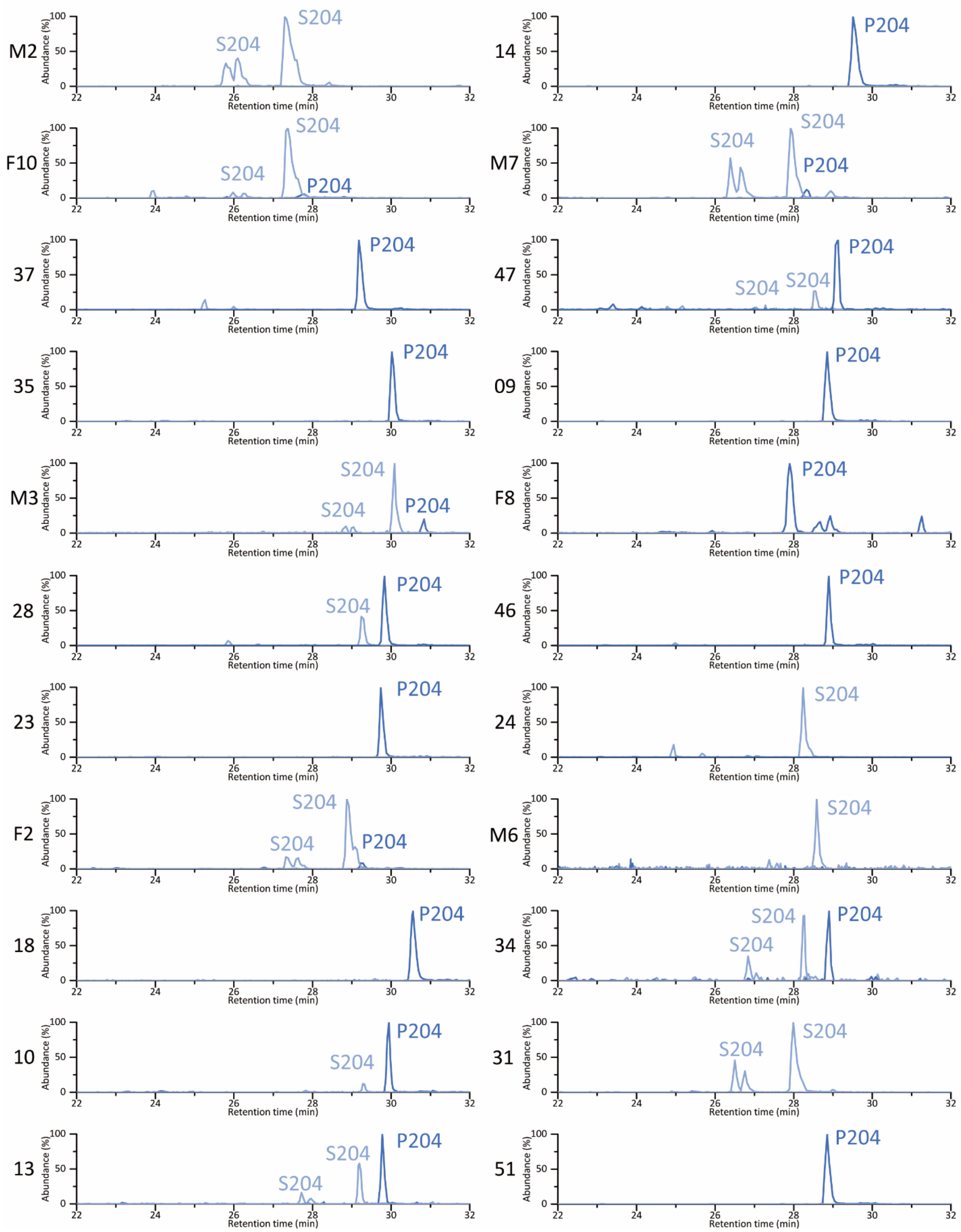

Supplementary Figure 4. LC-MS traces of unique allele specific peptides detected in HRG from the last 22 donors in order of measurement. Skyline was used for allele classification and quantification of the Pro204Ser substitution. Dark blue: Pro204, light blue: Ser204. The multiple peaks seen at Ser204 are glycoforms of the same peptide. The number on the left of y-axis provides the donor code.

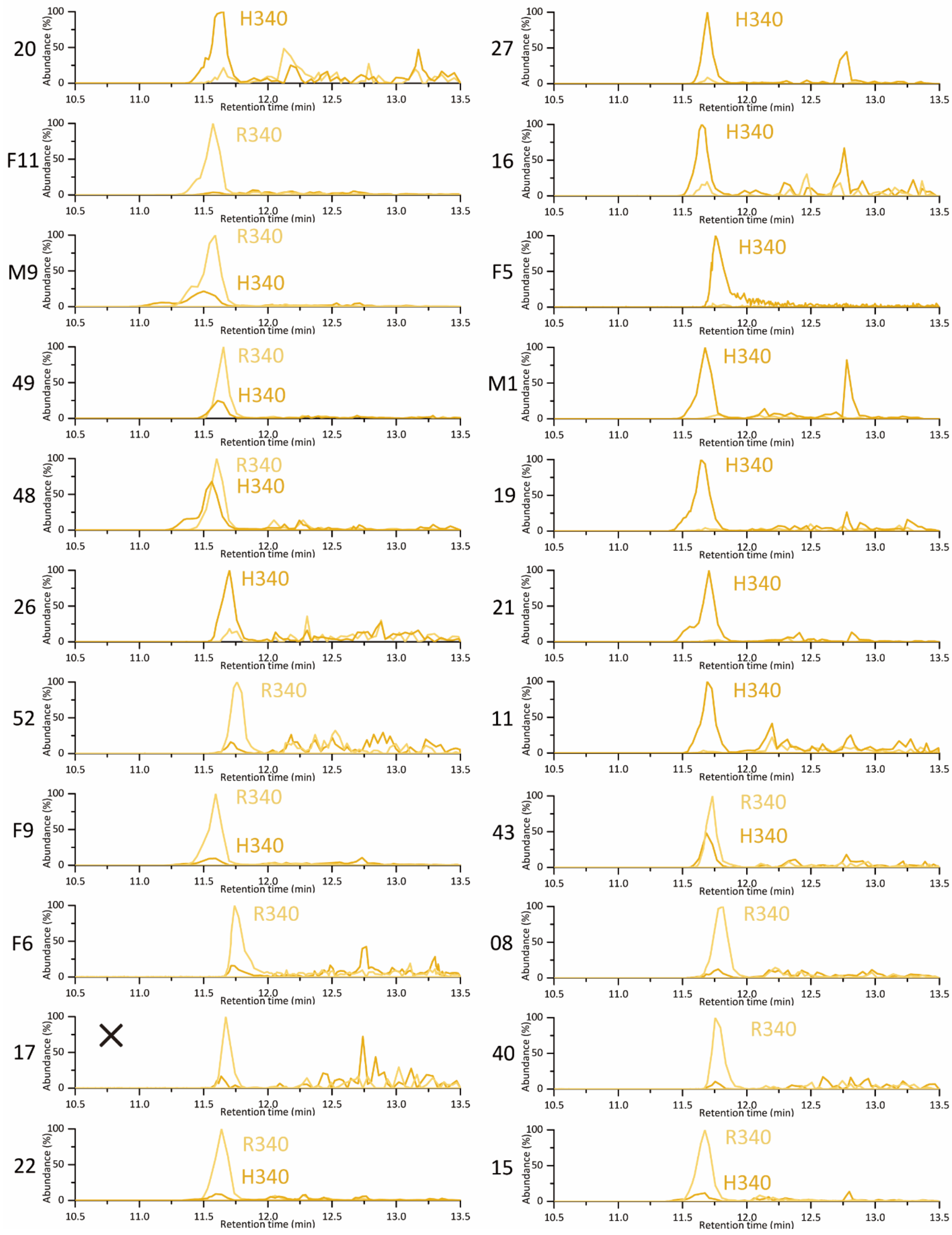

Supplementary Figure 5. LC-MS traces of unique allele specific peptides detected in HRG from the first 22 donors in order of measurement. Skyline was used for allele classification and quantification of the His340Arg substitution. Dark yellow: His340, light yellow: Arg340. The samples with black crosses are not assigned as alleles, either due to low intensities or mismatches with expected isotope ratios. The number on the left of y-axis provides the donor code.

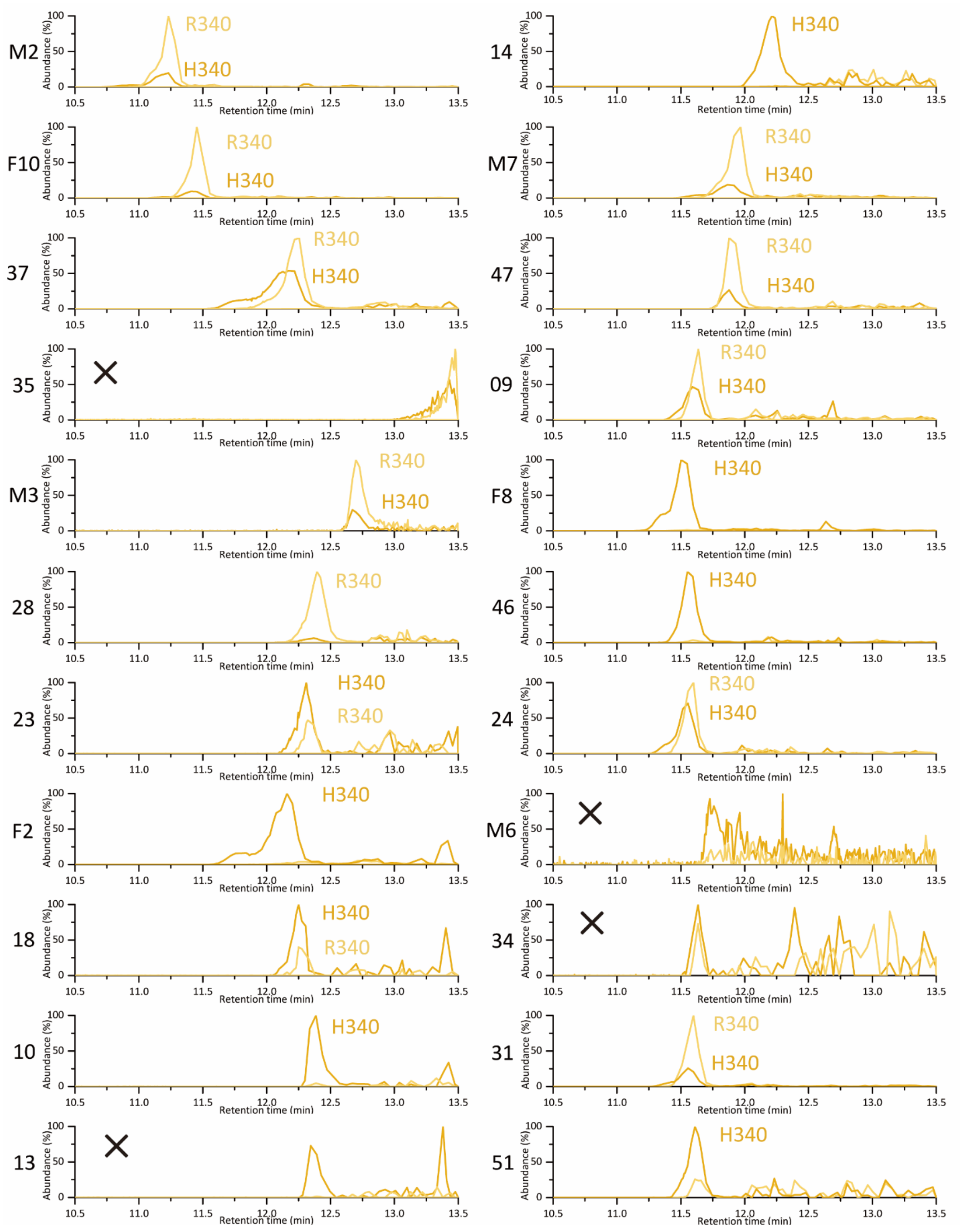

Supplementary Figure 6. LC-MS traces of unique allele specific peptides detected in HRG from the last 22 donors in order of measurement. Skyline was used for allele classification and quantification of the His340Arg substitution. Dark yellow: His340, light yellow: Arg340. The number on the left of y-axis provides the donor code. The samples with black crosses are not assigned as alleles, either due to low intensities or mismatches with expected isotope ratios.

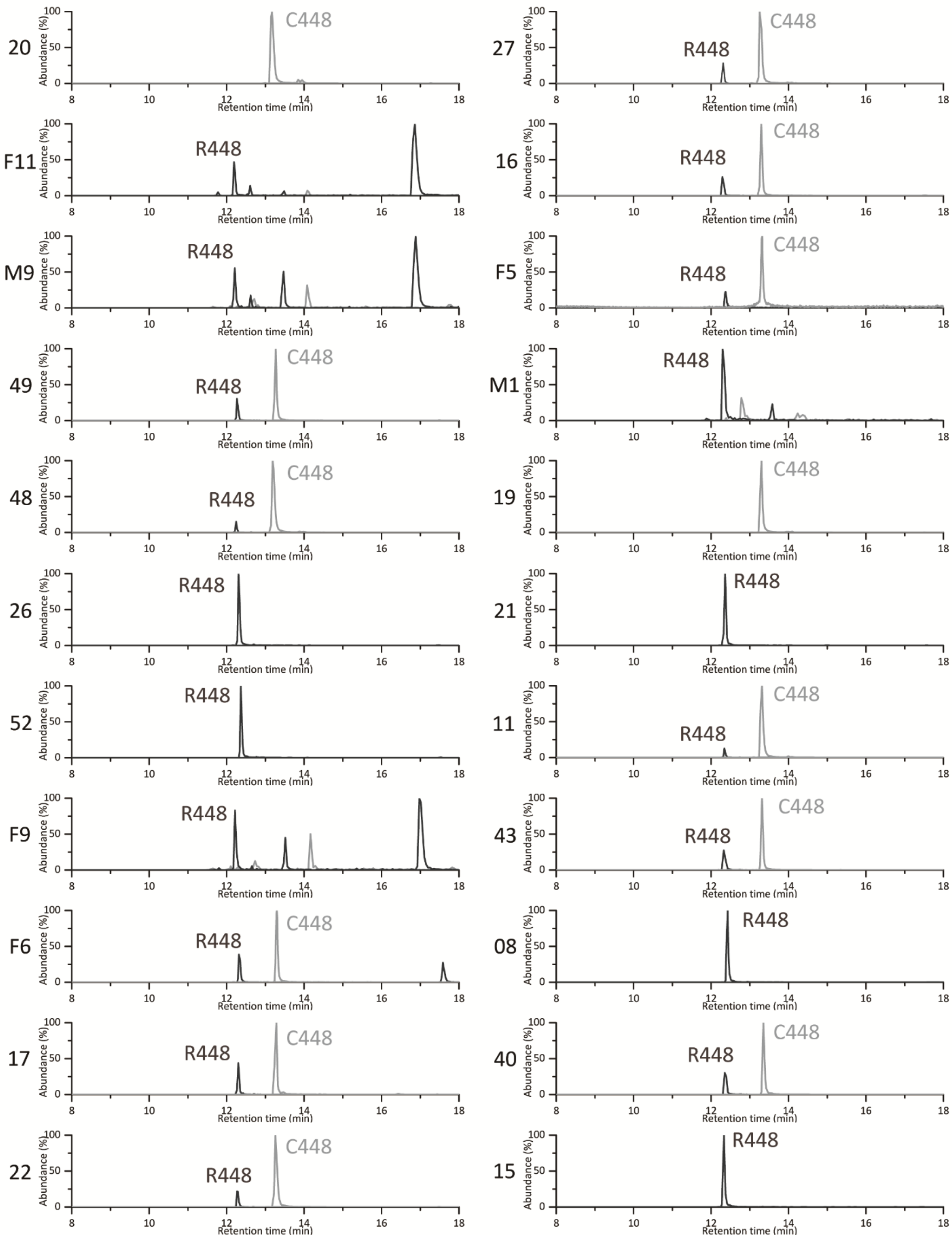

Supplementary Figure 7. LC-MS traces of unique allele specific peptides detected in HRG from the first 22 detected donors in order of measurement. Skyline was used for allele classification and quantification of the Arg448Cys substitution. Dark grey: Arg448, light grey: Cys448. The number on the left of y-axis provides the donor code.

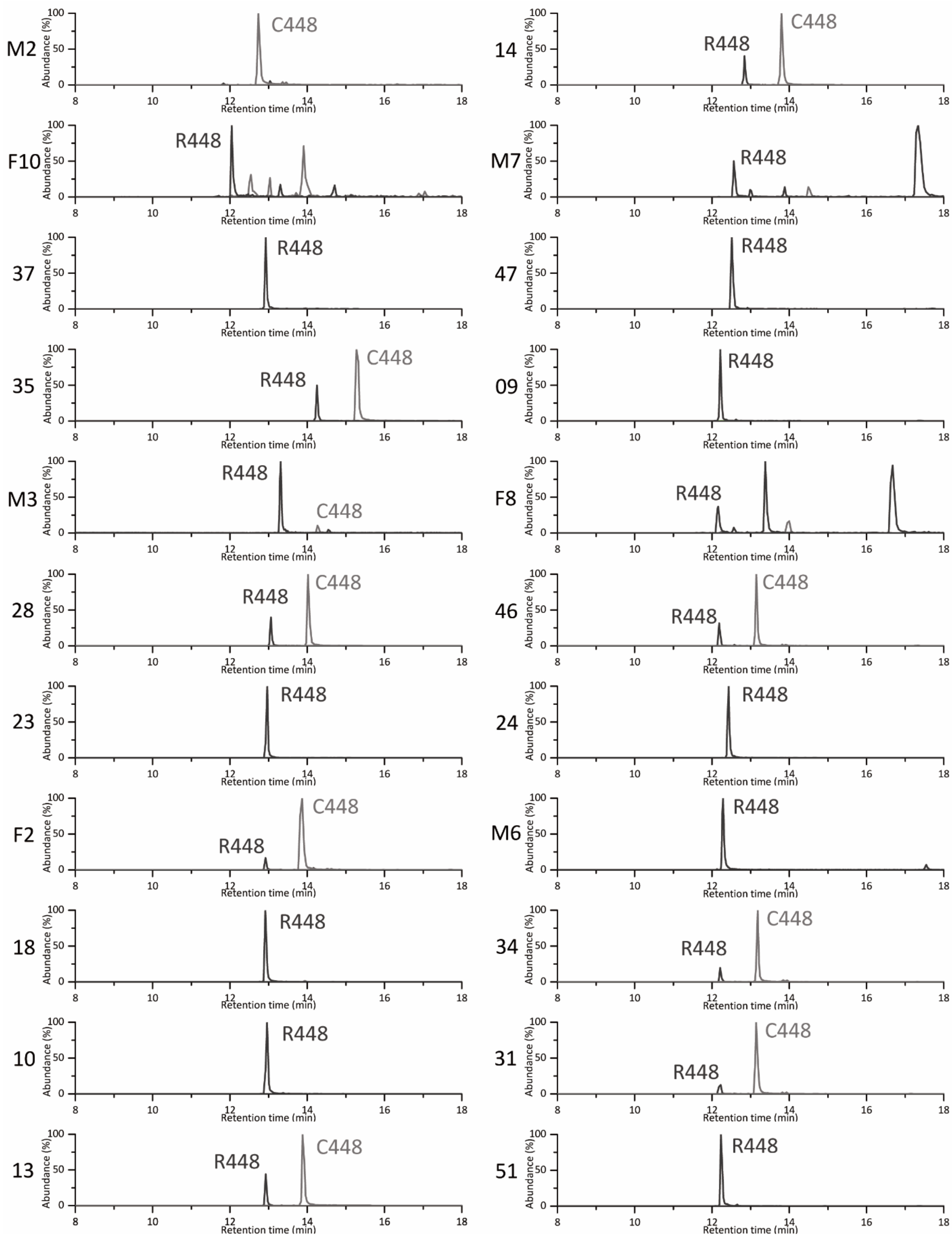

Supplementary Figure 8. LC-MS traces of unique allele specific peptides detected in HRG from the last 22 donors in order of measurement. Skyline was used for allele classification and quantification of the Arg448Cys substitution. Dark grey: Arg448, light grey: Cys448. The number on the left of y-axis provides the donor code.

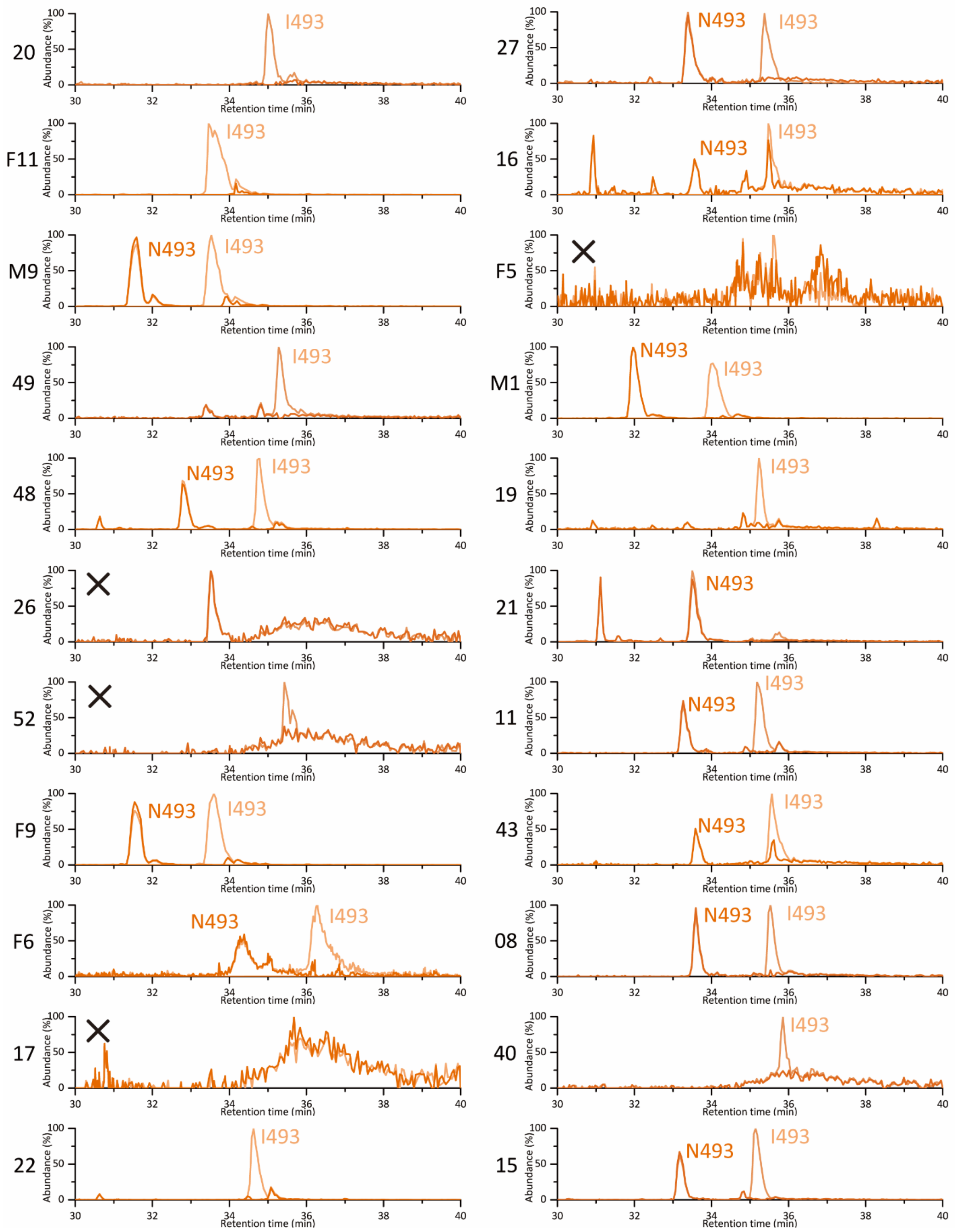

Supplementary Figure 9. LC-MS traces of unique allele specific peptides detected in HRG from the first 22 donors in order of measurement. Skyline was used for allele classification and quantification of the Asn493Ile substitution. Dark orange: Asn493, light orange: Ile493. The number on the left of y-axis provides the donor code. The samples with black crosses could not be assigned, either due to low intensities or mismatches with expected isotope ratios.

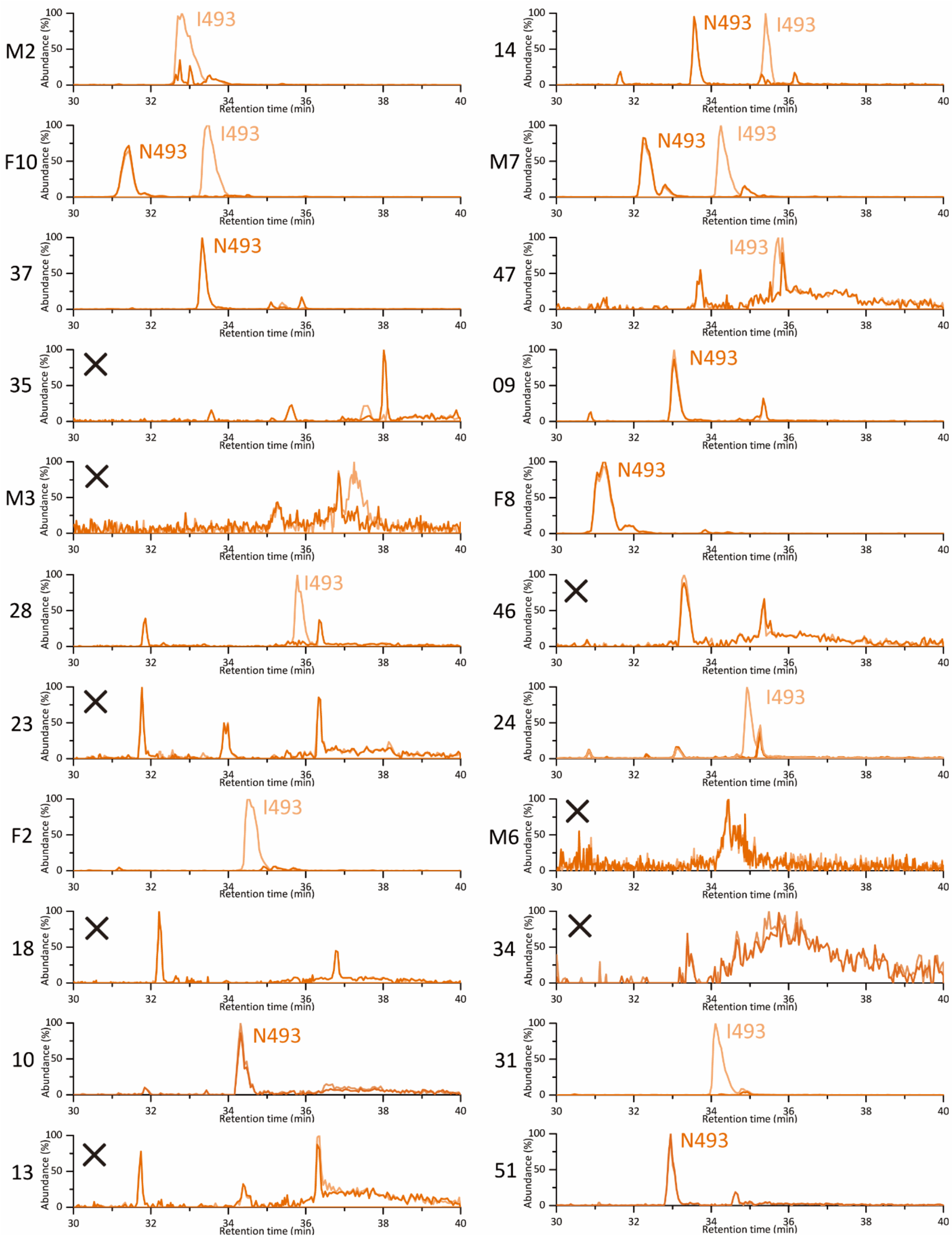

Supplementary Figure 10. LC-MS traces of unique allele specific peptides detected in HRG from the last 22 donors in order of measurement. Skyline was used for allele classification and quantification of the Asn493Ile substitution. Dark orange: Asn493, light orange: Ile493. The number on the left of y-axis provides the donor code. The samples with black crosses could not be assigned, either due to low intensities or mismatches with expected isotope ratios.

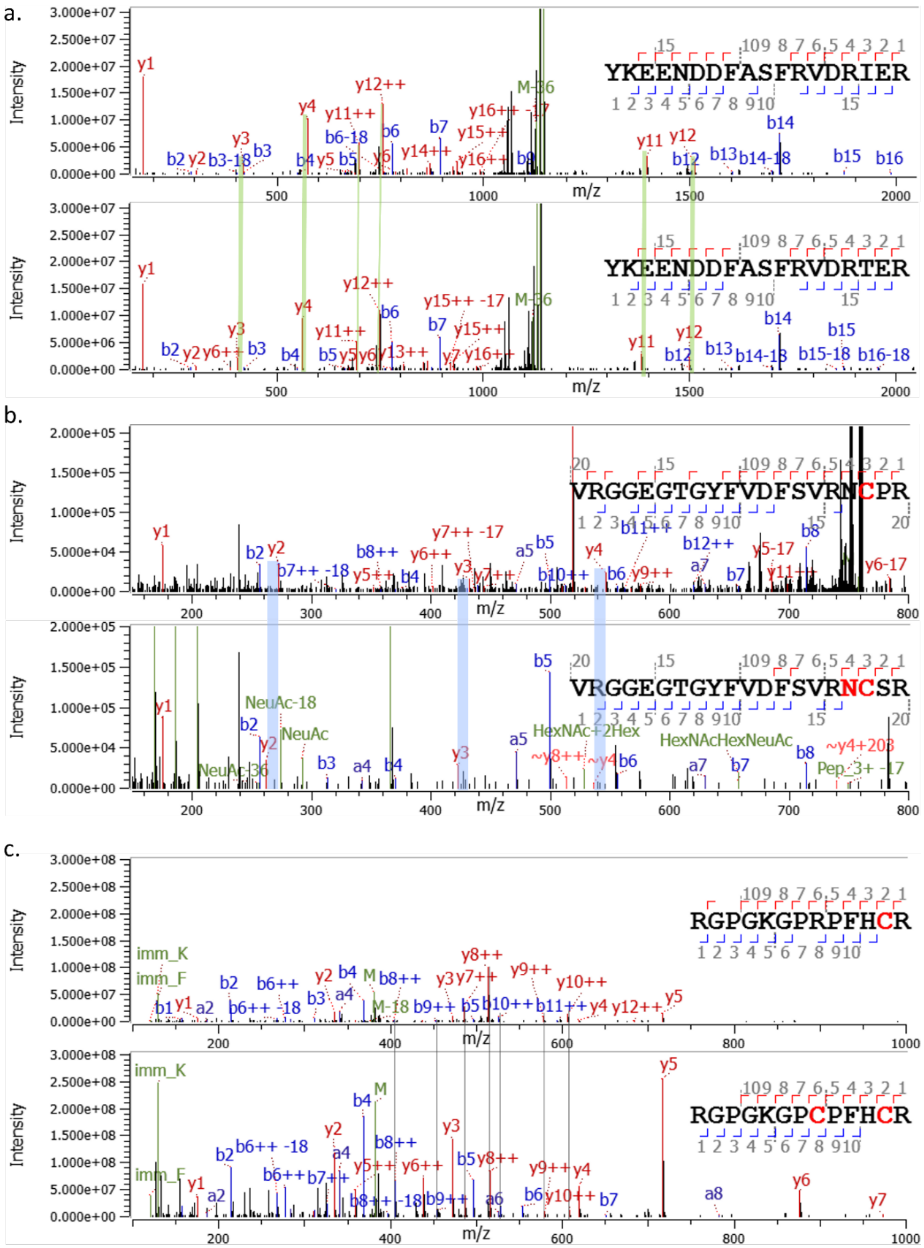

Supplementary Figure 11. MS/MS spectra illustrative for the unique peptides carrying the distinctive mutations in HRG. Annotated MS2 spectra of peptides covering **a)** the Ile180Thr substitution, **b)** the Pro204Ser substitution and **c)** the Arg448Cys substitution. The semi-transparent green, blue and gray lanes illustrate several of the ions that differ between the respective substitutions. The position of the mutation sites and fragment ion IDs and mass shifts are shown on the right. Fragmentation of these tryptic peptides was achieved by HCD.

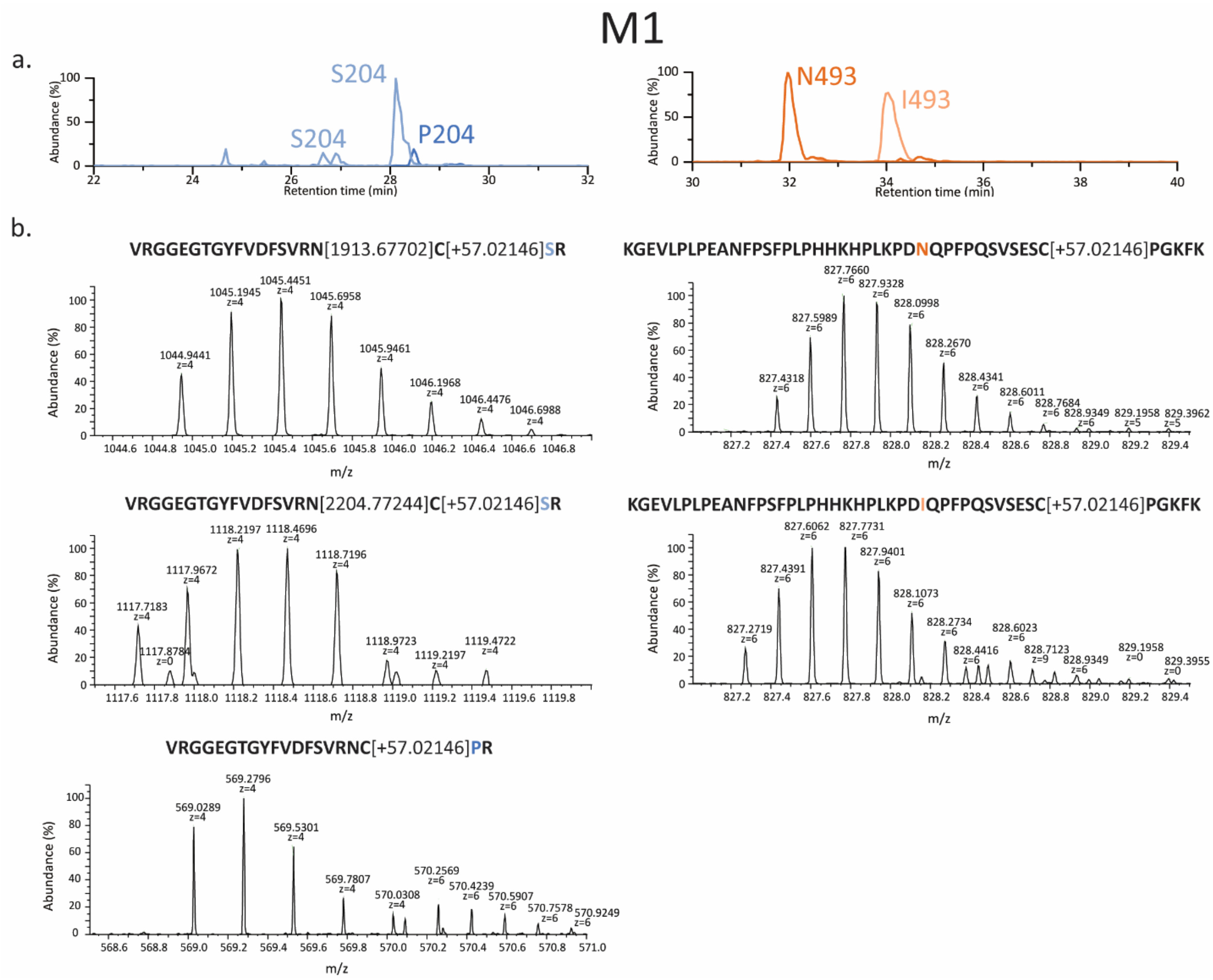

Supplementary Figure 12. Overview of the spectra from sample M1 with pairwise co-occurrences of allele-specific mutations SS204_NI493. a) LC-MS traces of unique allele specific peptides detected in HRG by Skyline used for allele classification and quantification of the mutations. **b)** MS1 spectra of unique allele specific peptides detected in HRG. Dark blue: Pro204, light blue: Ser204, dark orange: Asn493, light orange: Ile493. The modifications with the masses of 1913.67702 Da and 2204.77244 Da represent the glycans N4H5S1 and N4H5S2, respectively. The modifications with the masses of 57.02146 Da represent cysteine carbamidomethylation.

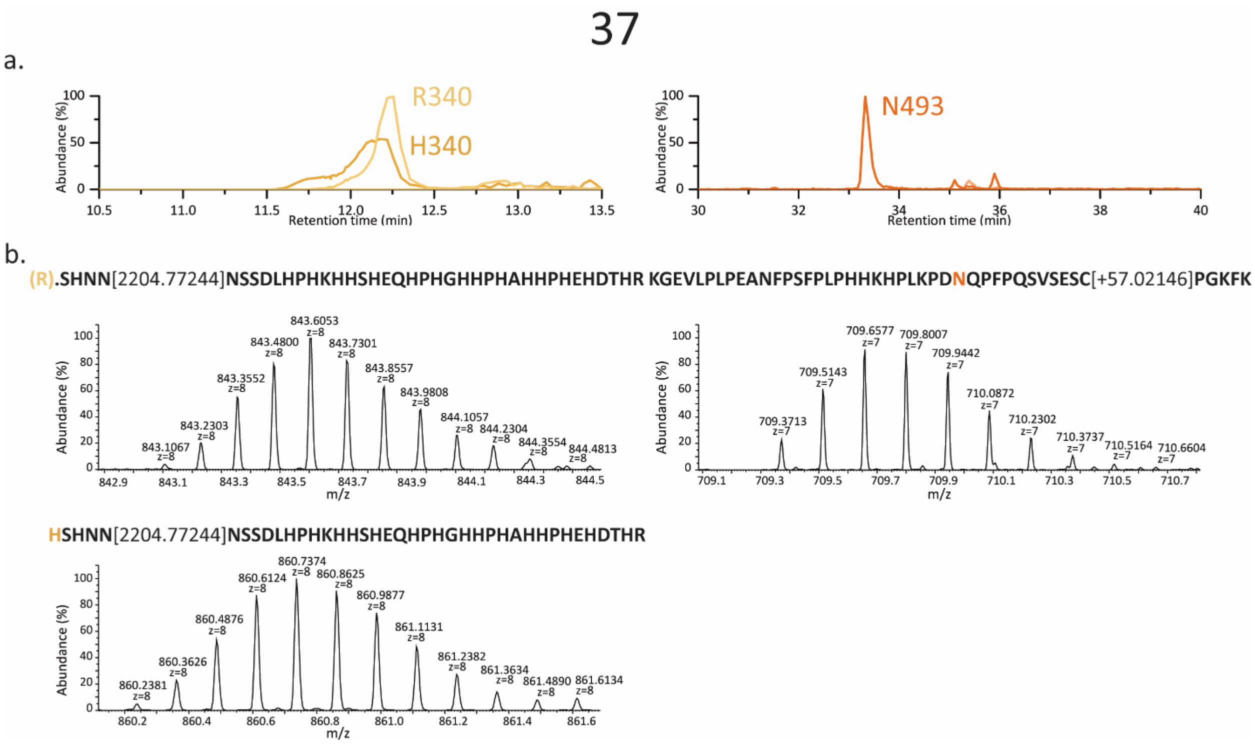

Supplementary Figure 13. Overview of the spectra from sample 37 with pairwise co-occurrences of allele-specific mutations HR340_NN493. a) LC-MS traces of unique allele specific peptides detected in HRG by Skyline used for allele classification and quantification of the mutations. **b)** MS1 spectra of unique allele specific peptides detected in HRG. Dark yellow: His340, light yellow: Arg340, dark orange: Asn493. The modifications with the mass of 2204.77244 Da represents the glycan N4H5S2, which is the most abundant glycan detected at the *N*-glycosylation site Asn344. The modification with the masses of 57.02146 Da represents cysteine carbamidomethylation.

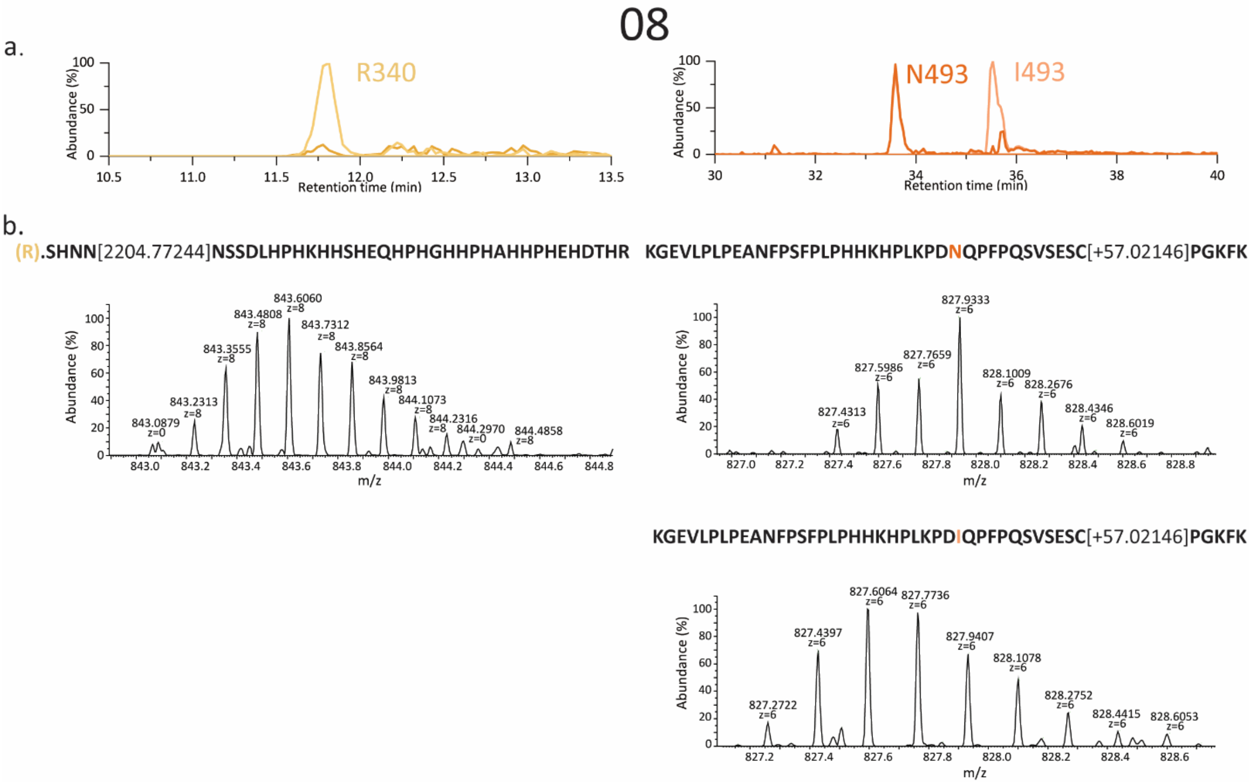

Supplementary Figure 14. Overview of the spectra from sample 08 with pairwise co-occurrences of allele-specific mutations RR340_NI493. a) LC-MS traces of unique allele specific peptides detected in HRG by Skyline used for allele classification and quantification of the mutations. **b)** MS1 spectra of unique allele specific peptides detected in HRG. Light yellow: Arg340, dark orange: Asn493, light orange: Ile493. The modification with the masses of 57.02146 Da represents cysteine carbamidomethylation.

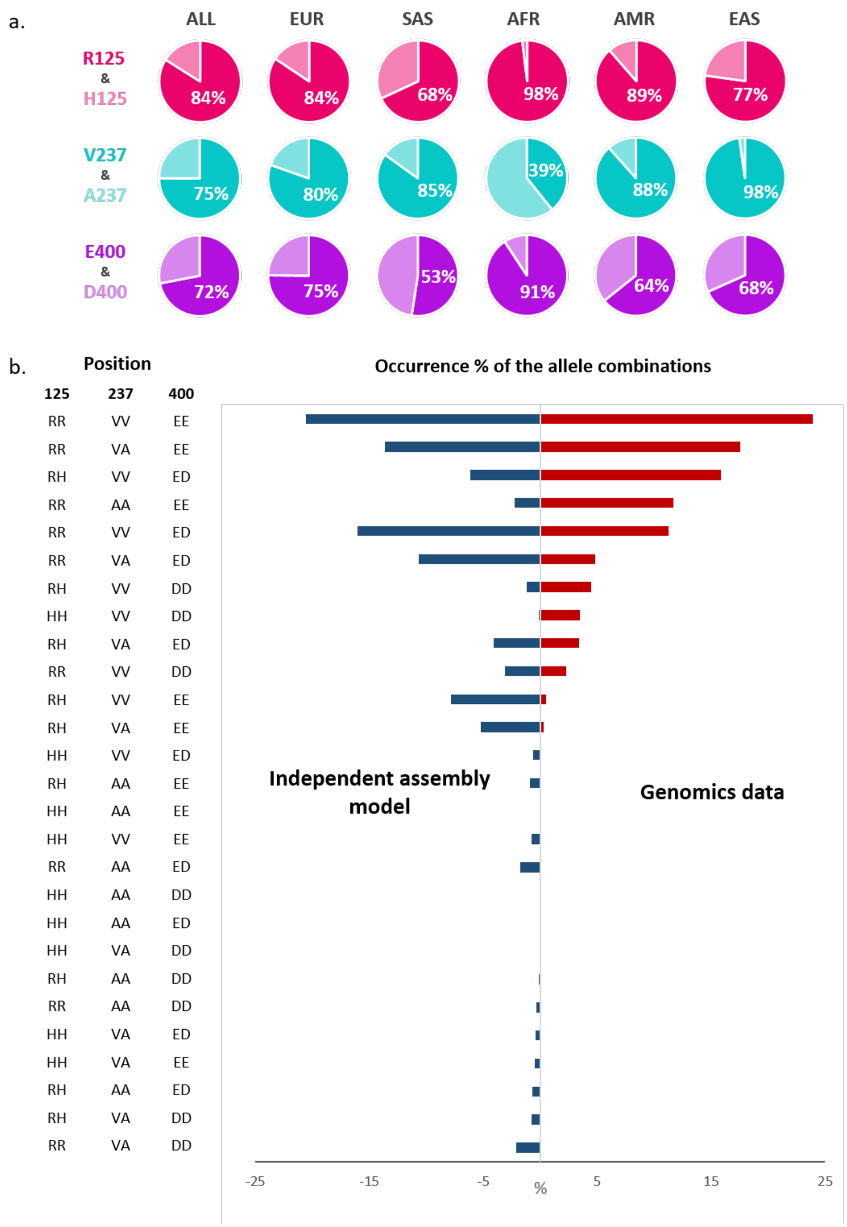

Supplementary Figure 15. Theoretical (independent assembly) and experimental (1000 genome data) co-occurrences of 3 allele frequencies in A1AT. a) The frequencies of three abundant gene variants of HRG in different subpopulations. The subpopulations are named as follows: EUR: European, SAS: south Asian, AFR: African, AMR: American, EAS: east Asian. **b**) Occurrence % of all combinations based on an independent assembly model (left) and the experimental 1000 genome data (right). The y-axis depicts the combinations of most frequent gene variants within HRG, with pairwise the amino acids at position 125, 237 and 400.

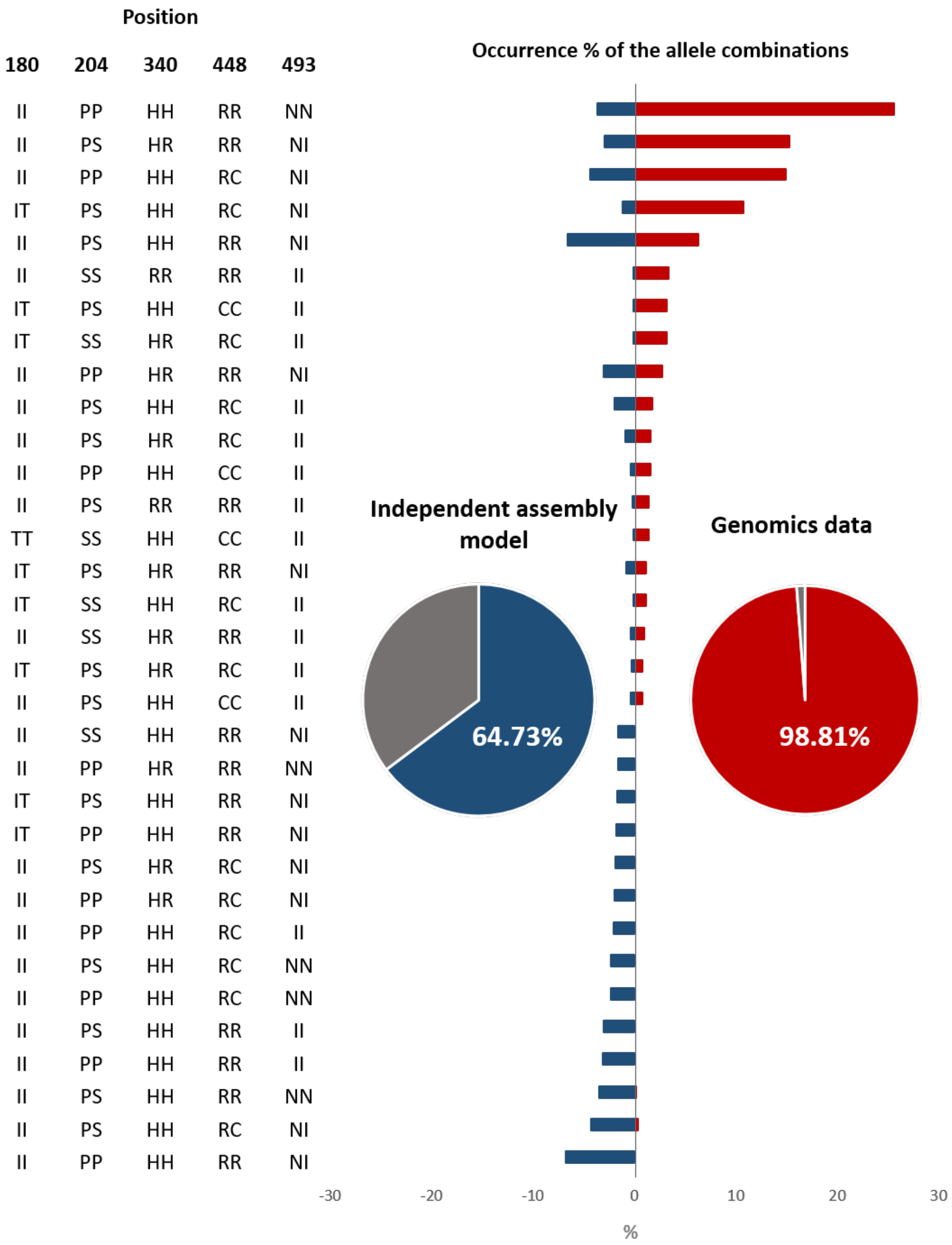

Supplementary Figure 16. Theoretical (European independent assembly) and experimental (503 Europeans from 1000 genome data) co-occurrences of 5 allele frequencies in HRG. Occurrence (%) of the top 20 abundant combinations based on a European independent assembly model (left) and the 503 Europeans from the experimental 1000 genome data (right). The y-axis depicts the combinations of most frequent gene variants within HRG, with pairwise the amino acids at position 180, 204, 340, 448 and 493.

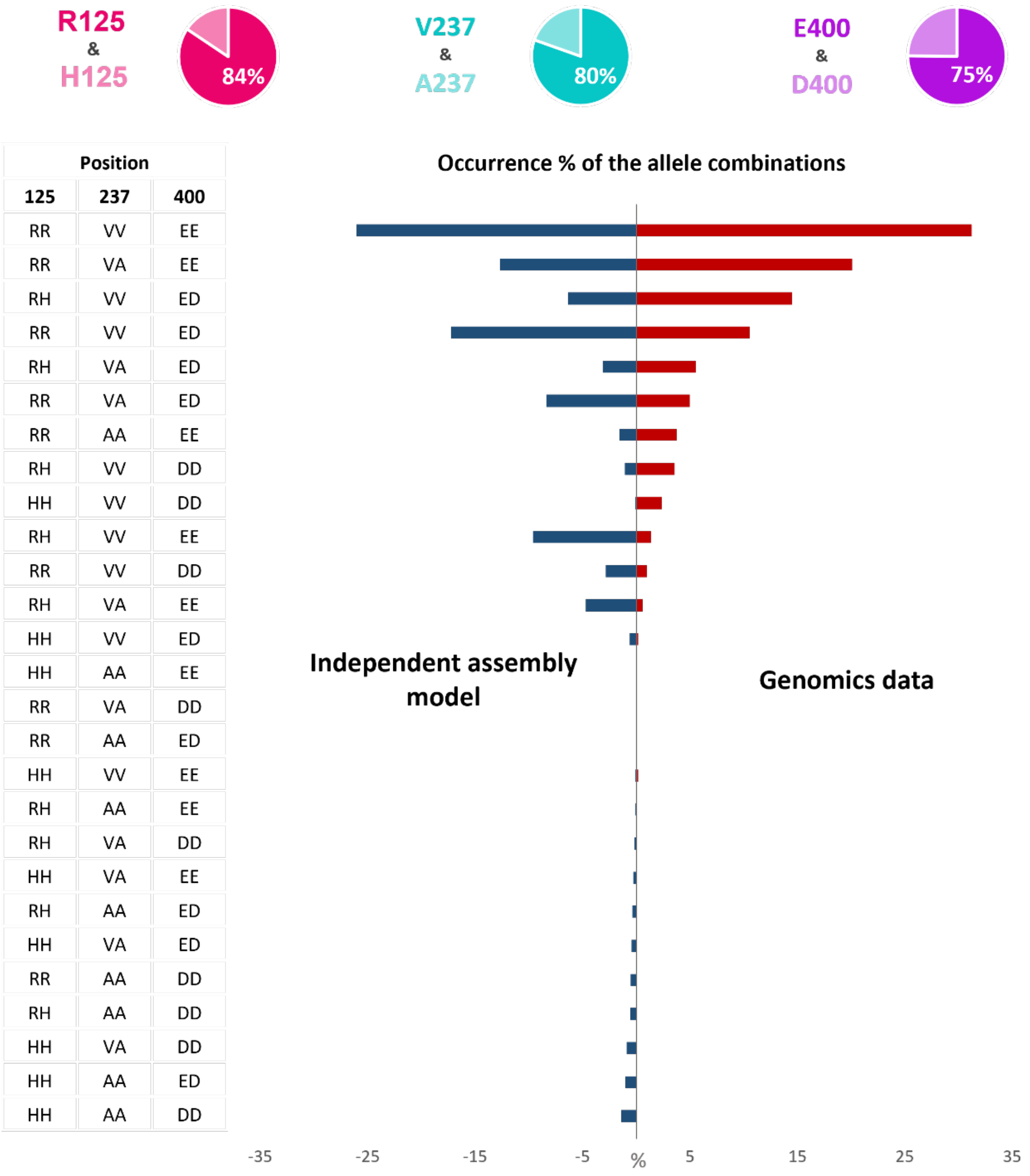

Supplementary Figure 17. Theoretical (European independent assembly) and experimental (503 Europeans from 1000 genome data) co-occurrences of 5 allele frequencies in A1AT. The piechart shows the frequencies of 3 abundant gene variants of A1AT in European. Occurrence % of all combinations based on a European independent assembly model (left) and the 503 Europeans from the experimental 1000 genome data (right). The y-axis depicts the combinations of most frequent gene variants within HRG, with pairwise the amino acids at position 125, 237 and 400.

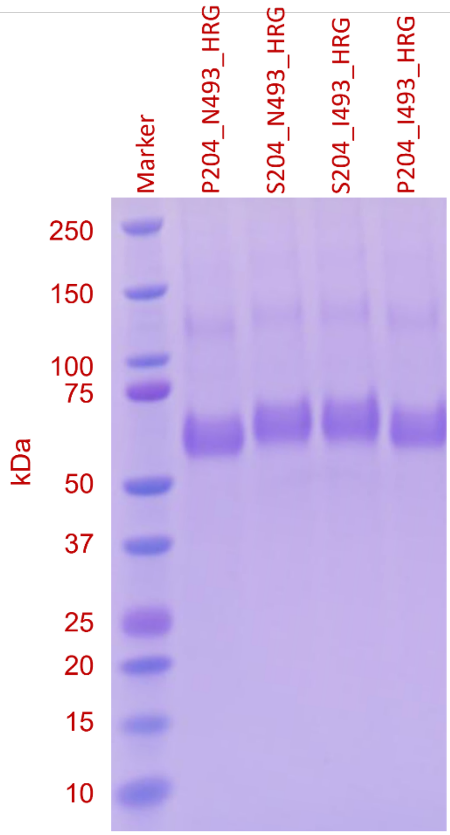

Supplementary Figure 18. Non-reducing gel of recombinant HRG proteins produced in and purified from HEK293 cells. The bands from the second lane to fifth lane are HRG with PP204_II493, PP204_NN493, SS204_II493, and SS204_NN493 respectively. For each lane, 15 µL protein was loaded.
